# Sequence-based comparative secretome analysis reveals conserved core effectors and host lineage-specific divergence between monocot- and dicot-associated powdery mildew lineages

**DOI:** 10.3389/fpls.2026.1783609

**Published:** 2026-03-09

**Authors:** Noman Ali, Nan Wu, Mahinur S. Akkaya

**Affiliations:** School of Bioengineering, Dalian University of Technology, Dalian, China

**Keywords:** effector proteins, host-associated divergence, orthogroup, powdery mildew fungi, secretome prediction

## Abstract

Powdery mildew fungi are obligate biotrophs that parasitize living plant tissues and deploy secreted proteins to support host colonization. We compared predicted secretomes from 26 powdery mildew isolates representing five genera (*Blumeria*, *Erysiphe*, *Golovinomyces*, *Parauncinula*, and *Podosphaera*) and encompassing monocot- and dicot-associated lineages. A standardized prediction and filtering workflow identified 7,545 secretome candidates from 219,897 proteins, which were then analyzed by orthogroup clustering, N-terminal motif screening, subcellular localization prediction, functional annotation, and homology searches against reported powdery mildew effectors. OrthoFinder assigned candidates to 1,399 orthogroups, revealing a conserved shared component across genera together with extensive genus- and isolate-specific diversification. Candidates were biased toward short mature proteins and were dominated by low-to-moderate cysteine ratios; higher cysteine content coincided with an increased proportion of proteins predicted to localize to the apoplast. N-terminal Y/F/WxC motifs were frequent in *Blumeria* secretomes and showed genus-specific positional preferences in mature sequences. At least one database-supported annotation was obtained for 4,148 candidates, with common categories including Egh16-like virulence factors, proteases, glycoside hydrolases, and ribonuclease-related annotations. Homology mapping of 75 known powdery mildew effectors identified conserved, high-abundance orthogroup-linked modules spanning multiple genera and *Blumeria*-restricted expansion modules. Proteome-wide searches further supported EqCmu and EqPdt as broadly conserved non-canonical (signal peptide-lacking) effectors with strong sequence and structural conservation across powdery mildew isolates.

## 1 Introduction

The powdery mildew fungi (*Erysiphaceae*) are widespread obligate biotrophic pathogens that infect over 10,000 plant species, including major monocot and dicot crops such as wheat, barley, cucumber, tomato, and grapevine, leading to significant economic losses (Glawe, 2008). Their strict dependency on living host tissue necessitates the establishment of a complex molecular interface, where secreted proteins, collectively termed the secretome, play pivotal roles by acting in the apoplast to modulate extracellular defenses or by reaching intracellular compartments to reprogram immunity and metabolism, thereby suppressing host immunity and facilitating nutrient acquisition (Barsoum et al., 2019; Bilstein-Schloemer et al., 2025). Consequently, characterizing the repertoire of secreted proteins, particularly effector proteins that directly modulate host plant physiology, is fundamental to understanding powdery mildew pathogenesis and host adaptation.

Over the past decade, powdery mildew effector biology has advanced rapidly, driven by expanded genome resources and methodological innovations that partially offset the experimental limitations of obligate biotrophy (Bindschedler et al., 2016; Kusch et al., 2024; Bilstein-Schloemer et al., 2025). Effector biology in powdery mildew fungi has advanced significantly through studies in model systems such as *Blumeria graminis* f. sp. *hordei* (barley powdery mildew), *Blumeria graminis* f. sp. *tritici* (wheat powdery mildew), and *Golovinomyces orontii* (*Arabidopsis* powdery mildew). In cereal-infecting *Blumeria graminis* lineages, large catalogs of candidate secreted effector proteins (CSEPs) have been defined and linked to haustorial expression, host genotype specificity, and immune modulation (Pedersen et al., 2012; Wicker et al., 2013; Bourras et al., 2018). These catalogs revealed characteristic sequence features in powdery mildew effectors, including enrichment of short, secreted proteins and the frequent occurrence of N-terminal Y/F/WxC motifs in *Blumeria*-associated candidates (Godfrey et al., 2010; Pedersen et al., 2012). In parallel, work in dicot-infecting powdery mildew systems has expanded the mechanistic landscape of powdery mildew virulence. For example, *Erysiphe necator* effector CSEP087 targets grapevine arginine decarboxylase VviADC to suppress ROS-associated immunity (Mu et al., 2022), a chitin-binding effector PxCDA3 of *Podosphaera xanthii* that sequesters immunogenic chitin oligomers to dampen chitin-triggered immunity (Martínez-Cruz et al., 2022) and *Erysiphe quercicola* effector Eae1 attenuates ethylene-mediated resistance by acting at chloroplasts via HbAIG1 and destabilizing HbSAMS5 (Shan et al., 2025). Notably, effector functions in powdery mildews are not exclusively mediated by “canonical” secreted proteins containing signal peptides. The discovery of non-canonical virulence factors like EqCmu, EqPdt, and EqIsc1 (He et al., 2021; Yin et al., 2024; Liu et al., 2025), which lack such signals, demonstrates that effector activity is independent of classical secretion pathways. These findings indicate that comprehensive effector catalogs will require searches that extend past canonical secretome boundaries.

Comparative secretome analysis has emerged as a powerful approach to elucidate conserved virulence strategies and lineage−specific adaptations among fungal pathogens (Lo Presti et al., 2015; Jia et al., 2023). In powdery mildews, this approach is particularly informative because their obligate biotrophic lifestyle is coupled to pronounced genome remodeling, including genome expansion driven by transposon proliferation and extensive gene loss, alongside rapid diversification and turnover of putative effector families (Spanu et al., 2010; Menardo et al., 2017). Such rapid genomic dynamics are consistent with the strong and continuous selection imposed by host specialization and immune surveillance. This suggests that powdery mildew secretomes are shaped by two complementary evolutionary forces: (i) the maintenance of a set of conserved effectors essential for core biotrophic functions, and (ii) extensive lineage−restricted expansions that reflect host association and evolutionary history (Liang et al., 2018; Wu et al., 2018).

Despite this progress, a coherent cross-genus view of secretome diversification in powdery mildews remains limited. Effector discovery and functional validation are concentrated in a small number of models pathosystems, especially cereal-infecting *Blumeria*, which makes it harder to define conserved core modules across the broader *Erysiphaceae* and can bias general conclusions toward *Blumeria*-linked signatures (Barsoum et al., 2019; Bilstein-Schloemer et al., 2025). Comparative genomics has provided clear hints of host-lineage contrasts: monocot-associated powdery mildews tend to show larger and more rapidly expanding CSEP families with stronger signals consistent with positive selection, whereas dicot-associated lineages show fewer CSEP families and patterns consistent with stronger purifying selection and contraction (Liang et al., 2018; Wu et al., 2018). However, direct cross-study comparisons remain problematic because predicted secretomes have typically been generated using non-uniform pipelines and filtering criteria, limiting comparability across taxa and datasets. Additionally, effector discovery has largely relied on canonical secretion signals, which can overlook signal peptide-lacking effectors such as EqCmu and EqPdt (He et al., 2021; Liu et al., 2025). Together, these issues argue for a standardized analysis across a broad taxonomic range to identify a conserved core and lineage-restricted diversification patterns in powdery mildew secretomes.

To address these limitations, predicted secretomes from 26 powdery mildew isolates across five genera (*Blumeria*, *Erysiphe*, *Golovinomyces*, *Parauncinula*, and *Podosphaera*) were analyzed, spanning monocot- and dicot-associated lineages. A standardized prediction and filtering pipeline was applied to generate comparable secretome candidate sets, followed by orthogroup clustering to quantify shared, genus-specific, and isolate-specific components. Effector-linked sequence features (mature length, cysteine ratio, and N-terminal Y/F/WxC motifs) were profiled, and subcellular localization predictions were integrated with functional annotations to summarize secretome composition. Homologs of reported powdery mildew effectors were mapped across isolates by BLASTP searches against predicted secretomes, and whole-proteome searches were additionally performed for non-canonical effectors lacking signal peptides. This integrated design resolves conserved, high-abundance effector modules and defines lineage-restricted diversification linked to monocot- versus dicot-associated powdery mildew lineages.

## 2 Materials and methods

### 2.1 Proteome collection and secretome prediction

Proteomes of 26 powdery mildew isolates representing diverse host specificities (monocot and dicot) were collected as detailed in **Supplementary Table 1**, which lists the source and corresponding references for each isolate. Most of them were downloaded from NCBI (https://www.ncbi.nlm.nih.gov/), the Joint Genome Institute Genome Portal (https://genome.jgi.doe.gov/portal/) (Nordberg et al., 2014), and one proteome was obtained from the Pérez-García laboratory (Polonio et al., 2021). Protein sequences were formatted in FASTA using TBtools-II (Chen et al., 2023) to facilitate downstream analyses. To assess the completeness and annotation quality of the proteomes used in this study, each dataset using BUSCO (Benchmarking Universal Single-Copy Orthologs) v6.0.0 with the fungi_odb10 lineage dataset was evaluated (Tegenfeldt et al., 2025). BUSCO scores for all isolates are reported in **Supplementary Table 1**. Redundant protein sequences (100% amino-acid identity) within each isolate were removed prior to secretome prediction, and the non-redundant set was used for downstream analyses. Signal peptides (SPs) were predicted using both SignalP v6.0 (Teufel et al., 2022) and Phobius (Käll et al., 2007), and only proteins identified as signal peptide−positive by both tools were retained, with the cleavage sites determined by SignalP v6.0 used to define mature sequences. Proteins containing transmembrane domains, identified by DeepTMHMM v1.0 (Hallgren et al., 2022) and InterProScan v107.0 (Blum et al., 2025), or carrying glycosylphosphatidylinositol (GPI) anchors predicted by NetGPI v1.1 (Gíslason et al., 2021), were excluded to generate a set of secretome candidates (predicted secretome). Mature protein sequences were defined by removing the SP prior to downstream analyses. The length and cysteine content of mature proteins were calculated for all secretome candidates.

### 2.2 Phylogenetic inference and orthogroup analysis

A species phylogeny was inferred from single-copy orthologous genes identified by OrthoFinder v2.5.5 (Emms and Kelly, 2019) using the entire proteomes of the 26 powdery mildew isolates with default parameters and visualized with iTOL v7 (Letunic and Bork, 2024). To classify the evolutionary relationships among the secreted proteins, orthogroups (OGs) of secretome candidates were also identified using OrthoFinder v2.5.5 (Emms and Kelly, 2019) with default parameters. Principal component analysis (PCA) was performed using OECloud tools (https://cloud.oebiotech.com/) based on an orthogroup count matrix, where rows represent orthogroups, columns represent isolates, and each cell indicates the number of secretome candidates assigned to a given orthogroup in a given isolate. The first two principal components (PC1 and PC2) were used to generate a 2D scatter plot, illustrating patterns of similarity and differences among isolates.

### 2.3 N-terminal Y/F/WxC motif detection and subcellular localization prediction

N-terminal Y/F/WxC motifs were identified by applying custom regular expressions to the first 30 amino acids of mature protein sequences according to the previous study (Godfrey et al., 2010). Y/F/WxC motifs are defined as short N-terminal patterns in which a tyrosine (Y), phenylalanine (F), or tryptophan (W) residue is followed by any amino acid (x) and a conserved cysteine (C). Only the first occurrence of the motif was recorded. Subcellular localization predictions were performed using ApoplastP (Sperschneider et al., 2018), EffectorP v3.0 (Sperschneider and Dodds, 2022), DeepLoc v2.1 (Ødum et al., 2024), LOCALIZER (Sperschneider et al., 2017), and WoLF PSORT (Horton et al., 2007).

### 2.4 Functional annotation of secretome candidates

Functional annotation of mature protein sequences was performed using InterProScan v107.0 (Blum et al., 2025), integrating databases including CDD v3.2 (Wang et al., 2023), FunFam (Das et al., 2015), Gene3D v4.3.0 (Lewis et al., 2018), PANTHER v19.0 (Thomas et al., 2022), Pfam v38.0 (Paysan-Lafosse et al., 2025), PRINTS v42.0 (Attwood et al., 2012), ProSitePatterns v2025_01 and ProSiteProfiles v2025_01 (Sigrist et al., 2013), SMART v9.0 (Letunic et al., 2021), and SUPERFAMILY v1.75 (Oates et al., 2015). CAZy (Drula et al., 2022) and ENZYME (Release of 15-Oct-2025) (Bairoch, 2000) annotations were obtained using eggNOG-mapper v2.1.12 (Cantalapiedra et al., 2021) with default parameters, while KEGG v116.0 pathway annotations were performed using KofamKOALA (Aramaki et al., 2020).

### 2.5 Similarity search of known powdery mildew effectors

A set of 75 known powdery mildew effectors was compiled (**Supplementary Table 2**), including effector name or ID, source species, full-length protein sequences, sequences without SPs, and key references. Among these, 69 canonical effectors with predicted SPs either predicted by SignalP v6.0 (Teufel et al., 2022) or confirmed as secreted in previous studies were compared to the mature protein sequences of secretome candidates using standard protein BLAST in NCBI (BLASTP) (Altschul et al., 1990; Johnson et al., 2008). Putative homologs were defined by E-value < 1e^−5^, query coverage ≥ 50%, and percent identity ≥ 30%. Six non-canonical effectors lacking predicted SPs (AVR_k1_, Bh9o9, ROPIP1, EqCmu, EqIsc1, EqPdt) were searched against both the secretome candidates and the full proteomes of the 26 powdery mildew isolates using the same criteria. All BLASTP analyses were performed using default parameters.

### 2.6 Sequence alignment and structure prediction

Multiple sequence alignments of EqCmu, EqPdt (He et al., 2021; Liu et al., 2025) and their homologs were generated using MUSCLE (Edgar, 2004) and visualized using ESPript v3.2.0 (Robert and Gouet, 2014). Protein structures were predicted using AlphaFold 3 online server (Abramson et al., 2024), with predicted template modeling (pTM) and predicted local distance difference test (pLDDT) scores used to assess model confidence. Structural similarity among EqCmu, EqPdt, and their homologs was assessed using US-Align (Zhang et al., 2022), with TM-score indicating the degree of structural resemblance. All predicted structures were visualized and edited in PyMOL (https://www.pymol.org/).

## 3 Results

### 3.1 Comparative secretome prediction reveals expanded secretomes in monocot-associated powdery mildew lineages

**Figure 1:**
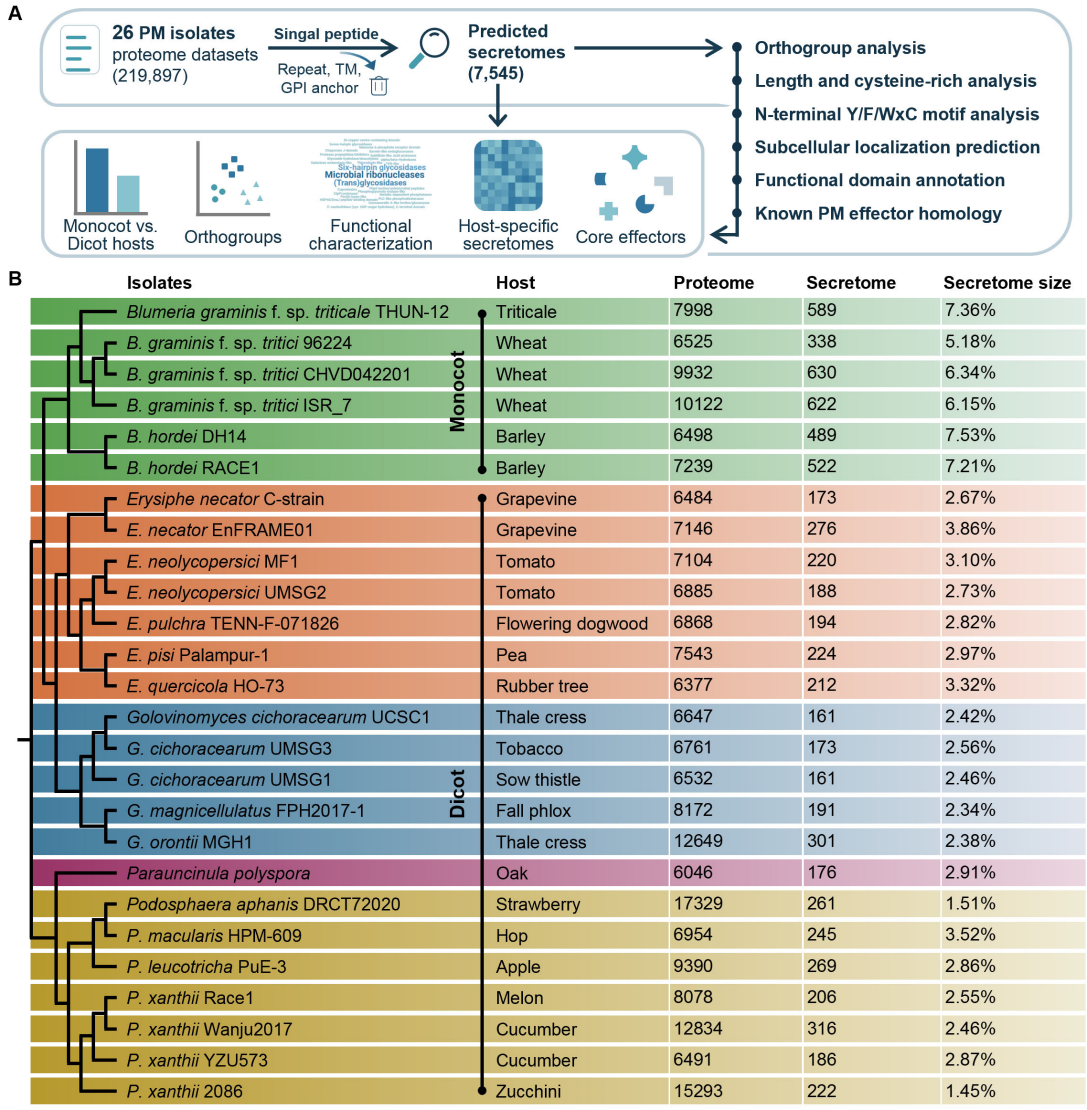
Overview of the secretome prediction pipeline and secretome statistics across 26 powdery mildew isolates. **(A)** Workflow of secretome identification and downstream analyses. Proteome datasets from 26 powdery mildew (PM) isolates were collected and filtered to remove redundant sequences. Proteins containing predicted N-terminal signal peptides were retained, while proteins with predicted transmembrane domains (TM) or glycosylphosphatidylinositol (GPI) anchor were excluded. The resulting set of 7,545 predicted secretome candidates was subjected to orthogroup analysis, sequence feature profiling (length, cysteine content, N-terminal Y/F/WxC motif), subcellular localization prediction, functional annotation, and homology searches against known powdery mildew effectors, enabling comparative analyses of monocot- and dicot-infecting species, host-specific secretomes, and core effector repertoires. **(B)** Phylogenetic relationships and predicted secretome statistics of the 26 powdery mildew isolates. A species phylogeny inferred from whole proteomes of 26 powdery mildew isolates is shown on the left. For each isolate, the host plant, number of predicted proteins (proteome), number of predicted secretome candidates (secretome), and the proportion of secretome proteins relative to the total proteome (secretome size) are summarized. Monocot- and dicot-infecting lineages are indicated.

To systematically characterize secretome diversity across powdery mildew fungi, we compiled predicted proteomes from 26 powdery mildew isolates representing five genera (*Blumeria*, *Erysiphe*, *Golovinomyces*, *Parauncinula*, and *Podosphaera*) with distinct host specificities, including monocot and dicot-infecting lineages (**Supplementary Table 1**). A unified secretome prediction workflow was applied to 219,897 protein sequences (**Figure 1A**). After redundancy removal within each isolate, proteins with a predicted N-terminal signal peptide and without transmembrane domains or glycosylphosphatidylinositol (GPI) anchor signals were retained as secretome candidates, yielding 7,545 predicted secreted proteins (**Supplementary Table 3**) for downstream comparative analyses.

Whole proteome phylogenetic analysis resolved clear genus-level clustering among the 26 powdery mildew isolates (**Figures 1B**, left). Proteome sizes varied substantially (6,046–17,329 proteins per isolate), and the number of predicted secretome candidates also varied widely (161–630; mean = 290) (**Figures 1B**, right). To assess potential effects of heterogeneous annotation quality across public sources, we quantified proteome completeness using BUSCO. BUSCO assessment confirmed high completeness (Complete BUSCOs: 88.5–99.1%) for 25 of the 26 proteomes, indicating that the observed variation in proteome and secretome size primarily reflects biological differences rather than major gaps in annotation (**Supplementary Table 1**). One exception was *Podosphaera leucotricha* PuE-3, whose proteome BUSCO completeness was markedly low (13.9%) despite the corresponding genome completeness being high (97.2%), consistent with prior genome-based BUSCO reporting for this isolate (Gañán et al., 2020).

Importantly, predicted secretome size did not simply scale with proteome size across isolates. When normalized to total proteome size, the proportion of predicted secreted proteins averaged 3.6% but ranged from 1.45% (*Podosphaera xanthii* 2086) to 7.53% (*Blumeria hordei* DH14), suggesting marked lineage-level differences in the composition of the predicted secretome. In this dataset, monocot infection is represented by *Blumeria* lineages, whereas dicot infection spans four genera (*Erysiphe*, *Golovinomyces*, *Parauncinula*, and *Podosphaera*); thus, monocot–dicot contrasts are partially coupled to genus-level divergence. *Blumeria* isolates consistently exhibited expanded secretomes (approximately 5.18–7.53% of their predicted proteomes), whereas dicot-infecting isolates generally harbored smaller secretomes (typically 1.45–3.86%) (**Figure 1B**). We hereafter refer to these patterns as monocot-infecting (*Blumeria*-linked) versus dicot-infecting secretome profiles, and in subsequent sections use orthogroup- and homology-based analyses to define conserved effector groups (homologs detected in at least 10 isolates) and to quantify lineage-associated divergence beyond overall secretome size differences (noting that monocot–dicot contrasts are partially confounded with genus in this dataset). Together, this uniformly processed dataset defines the comparative space for the remainder of the study, from expanded secretome profiles to orthogroup diversification, functional feature profiling, and homology-based identification of conserved effector families.

### 3.2 Orthogroup analysis reveals a conserved shared component and extensive genus- and isolate-specific diversification in powdery mildew secretomes

To examine sequence relationships among the 7,545 predicted powdery mildew secretome candidates from 26 isolates, we performed orthogroup clustering using OrthoFinder and identified 1,399 orthogroups (**Supplementary Table 4**). Orthogroup sizes were uneven, with a small number of highly populated groups contributing large numbers of candidates, including OG0001 (142 proteins), OG0002 (104 proteins), and OG0003 (91 proteins), followed by progressively smaller orthogroups.

**Figure 2:**
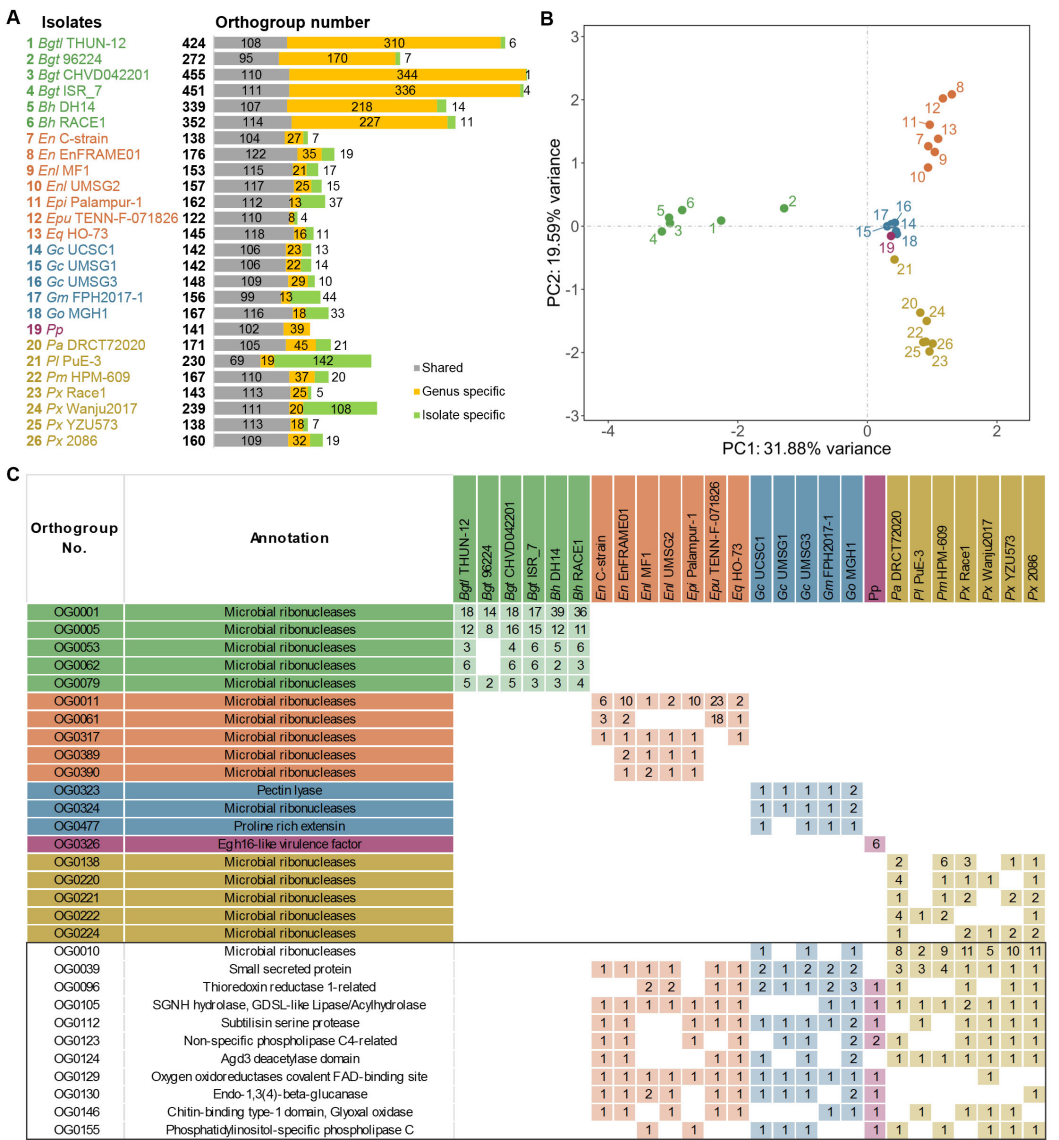
Orthogroup-based comparative analysis of secretome candidates from 26 powdery mildew isolates. **(A)** Distribution of shared (grey), genus-specific (orange), and isolate-specific (green) orthogroups across individual powdery mildew isolates. Orthogroups were formed using OrthoFinder based on 7,545 predicted secretome candidates. Shared orthogroups contain members from multiple genera, genus-specific orthogroups are restricted to a single genus, and isolate-specific orthogroups are unique to a single isolate. Bars indicate the number of orthogroups per category for each isolate. **(B)** Principal component analysis (PCA) based on the abundance matrix of secretome candidates per orthogroup across isolates. Each point represents one isolate, and clustering reflects similarities in orthogroup composition among secretomes. **(C)** Representative orthogroups with functional annotations showing genus-specific or host-associated distribution patterns. Selected orthogroups are enriched in secretome candidates and possess predicted functional annotations. Numbers indicate the count of secretome candidates per isolate within each orthogroup. Isolate names are shown using abbreviated labels as defined in **Supplementary Table 1**. The color coding of isolates in **(B, C)** corresponds to the isolate order and colors shown in **(A)**.

Orthogroup occupancy varied widely among isolates (122–455 orthogroups per isolate). Monocot-infecting *Blumeria* isolates consistently occupied the highest numbers of orthogroups (e.g., 455 in *B. graminis* f. sp. *tritici* CHVD042201 and 451 in *B. graminis* f. sp. *tritici* ISR_7), consistent with the expanded secretome profiles described in Section 3.1. To quantify conservation and specificity, orthogroups were classified by taxonomic composition into shared (members from ≥2 genera), genus-specific (restricted to a single genus), and isolate-specific (restricted to a single isolate) categories (**Figure 2A**). Shared orthogroup counts were relatively consistent across isolates (69–122 per isolate), indicating a conserved set of shared orthogroups maintained across powdery mildew genera. Notably, genus-specific orthogroups showed the strongest lineage-restricted signal in *Blumeria*, where per-isolate counts (170–344) were substantially higher than those of other genera (typically <45 per isolate). This prominent genus-restricted contribution in *Blumeria* represents one of the clearest patterns in the orthogroup composition analysis. Isolate-specific orthogroups were more variable and were elevated in several dicot-infecting isolates, including *Podosphaera leucotricha* PuE-3 (142) and *P. xanthii* Wanju2017 (108), indicating substantial isolate-level diversification in these lineages (**Figure 2A**).

To summarize compositional similarity among secretomes, we performed principal component analysis using the orthogroup abundance matrix in **Supplementary Table 4**, in which each value represents the number of secretome candidates assigned to a given orthogroup in a given isolate (**Figure 2B**). The PCA separated isolates into genus-level clusters, and the first principal component (PC1) distinguished *Blumeria* from dicot-infecting genera, indicating that orthogroup abundance profiles capture major axes of secretome divergence across powdery mildew fungi.

**Figure 2C** highlights representative orthogroups selected from abundant, annotated genus-specific sets and from annotated dicot-infecting shared orthogroups excluding *Blumeria* to provide concrete examples of lineage-associated secretome modules; numbers indicate absolute candidate counts per isolate. Among the genus-specific examples, large orthogroups from *Blumeria*, *Erysiphe*, *Golovinomyces*, and *Parauncinula* were frequently annotated as microbial ribonucleases, whereas *Podosphaera* was represented by an orthogroup annotated as an Egh16-like virulence factor (OG0326). In addition, dicot-infecting shared orthogroups excluding *Blumeria* included annotated categories consistent with diverse secreted functions, such as small secreted proteins (OG0039), thioredoxin reductase 1-related proteins (OG0096), and protease or hydrolase families (e.g., subtilisin serine protease OG0112 and SGNH hydrolase/GDSL-like lipase OG0105). These examples are presented to illustrate genus-associated secretome modules and to motivate the broader functional profiling in subsequent sections.

Together, orthogroup composition analyses indicate that powdery mildew secretomes include a conserved shared orthogroup component across genera, superimposed on pronounced genus-specific diversification in monocot-associated *Blumeria* and variable isolate-specific diversification in several dicot-infecting lineages, providing an orthogroup framework for downstream analyses of conserved effector groups and lineage-associated divergence.

### 3.3 Secretome candidates exhibit characteristic length distributions and higher cysteine content associated with predicted apoplastic localization

**Figure 3:**
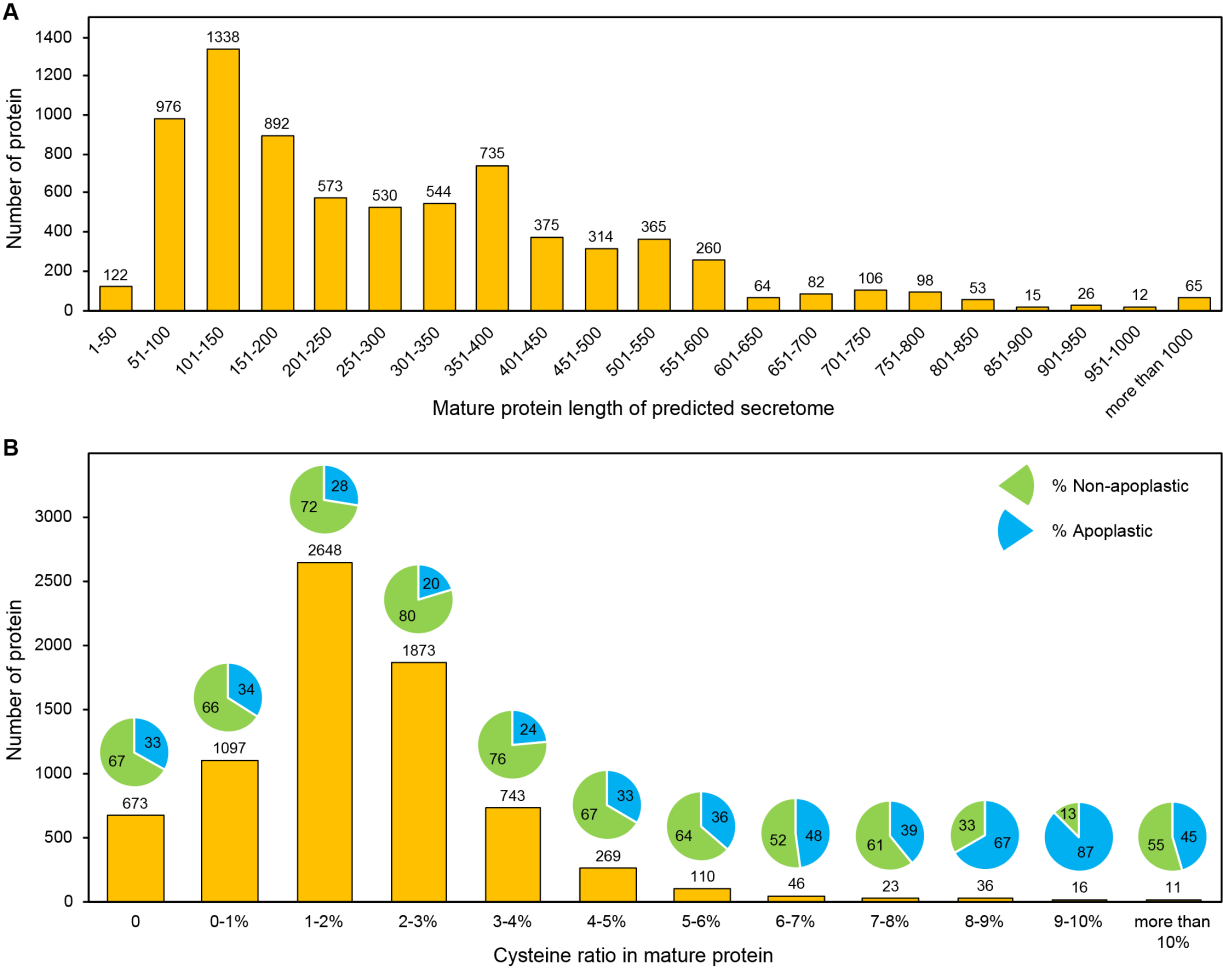
Length distribution and cysteine composition of secretome candidates from powdery mildew fungi. **(A)** Length distribution of mature protein sequences of 7,545 secretome candidates from 26 powdery mildew isolates after removal of predicted signal peptides. **(B)** Distribution of cysteine ratios in mature secreted proteins, calculated as the percentage of cysteine residues relative to total mature sequence length. Bars indicate the number of proteins in each cysteine ratio category, while pie charts show the proportions of non-apoplastic and apoplastic proteins predicted by ApoplastP.

To characterize sequence properties of the 7,545 predicted powdery mildew secretome candidates, we quantified mature protein length (after signal peptide removal) and cysteine ratio in mature proteins (**Figure 3**). Analysis of mature protein length revealed a predominance of shorter proteins among secretome candidates (**Figure 3A**). The largest number of candidates fell within the 101–150 amino acid range (1,338 proteins), followed by the 51–100 amino acid range (976 proteins) and the 151–200 amino acid range (892 proteins). Overall, 3,328 secretome candidates (44.1%) were 1–200 amino acids in length, and an additional 2,382 candidates (31.6%) were 201–400 amino acids. Consistently, 5,710 candidates (75.7%) were 400 amino acids or shorter. Longer secreted proteins were less frequent but were present across a wide range of lengths, including a small number exceeding 1,000 amino acids.

The cysteine ratio for each mature protein was calculated as the number of cysteine residues divided by mature sequence length and expressed as a percentage. Cysteine ratio analysis showed that most secretome candidates contained low to moderate proportions of cysteine residues (**Figure 3B**). A total of 2,648 proteins had cysteine ratios between 1% and 2%, and 1,873 proteins fell between 2% and 3%, together representing the most abundant cysteine classes. In total, 6,291 candidates (83.4%) had cysteine ratios of 3% or lower. Proteins with higher cysteine ratios were relatively uncommon; only 132 candidates exceeded 6% cysteine, indicating that high-cysteine proteins constitute a minor subset of the powdery mildew secretome. When predicted apoplastic localization was examined in relation to cysteine ratio, an increasing proportion of apoplast-predicted proteins was observed at higher cysteine ratios (**Figure 3B**). For candidates with cysteine ratios at or below 3%, approximately 20–34% were predicted to localize to the apoplast, whereas this proportion increased in higher cysteine categories, reaching 67% in the 8–9% range and 87% in the 9–10% range, despite the small number of proteins in these classes.

To test whether this pattern was lineage-restricted, we added a genus-stratified descriptive summary (**Supplementary Figure 1**). Because isolate-level high-cysteine bins often had zero or very small counts, isolates were combined by genus (*Blumeria*, *Erysiphe*, *Golovinomyces*, *Parauncinula*, and *Podosphaera*). Proteins were grouped into 0%, >0–6%, and >6% cysteine classes, and apoplastic/non-apoplastic proportions were summarized within each genus. Across analyzed genera, higher-cysteine classes generally showed higher apoplast-predicted proportions than lower-cysteine classes. This trend was observed beyond *Blumeria*, although its magnitude varied and high-cysteine bins were sparse in some groups.

Together, these results describe the distribution of protein length and cysteine composition among powdery mildew secretome candidates and show that higher cysteine content is associated with an increased likelihood of predicted apoplastic localization.

### 3.4 N-terminal Y/F/WxC motifs are more frequent and positionally biased in *Blumeria* secretomes

**Figure 4:**
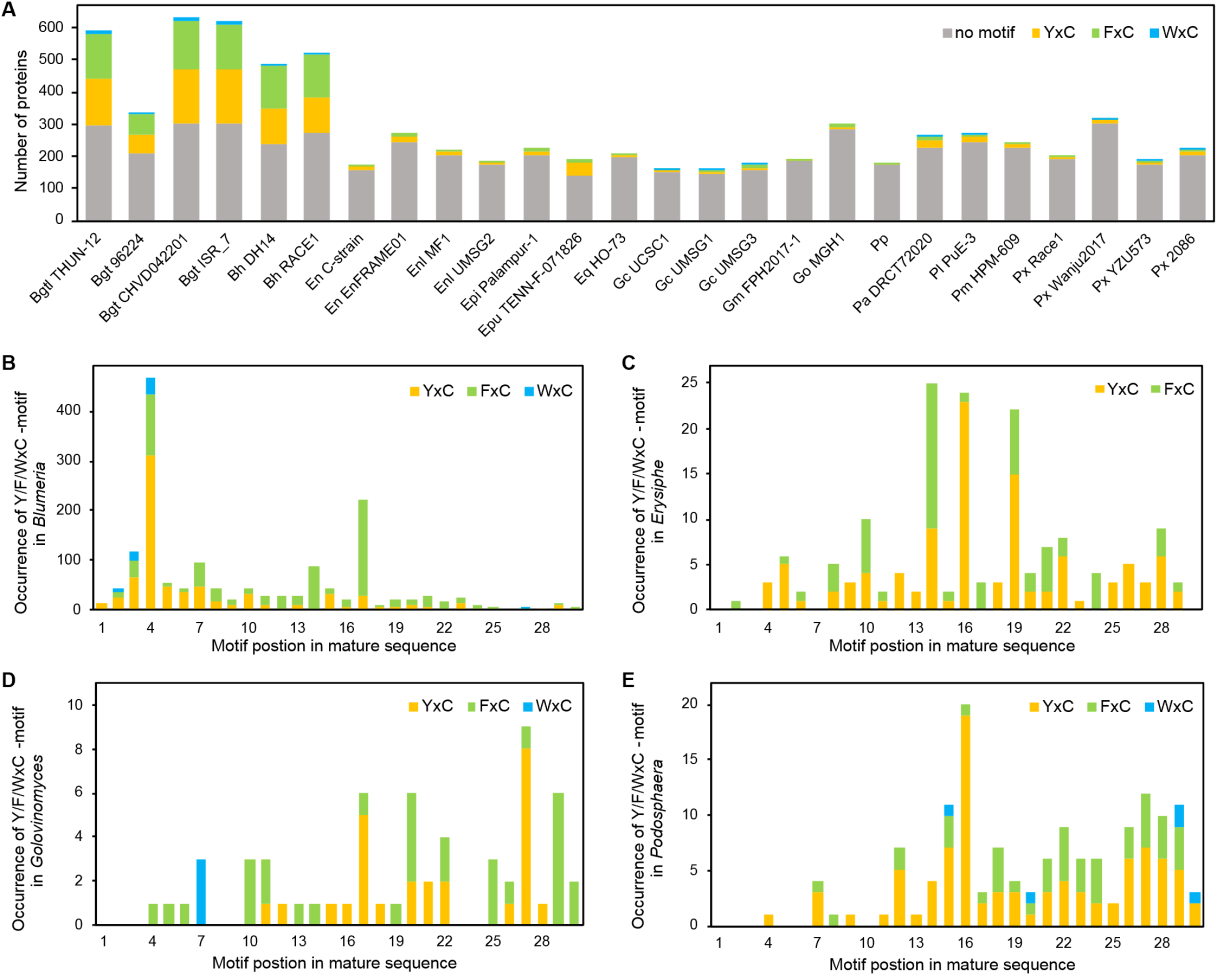
Distribution and positional occurrence of N-terminal Y/F/WxC motifs in the powdery mildew secretome. **(A)** Numbers of secretome candidates from 26 powdery mildew isolates containing YxC, FxC, or WxC motifs, or lacking any of these motifs, within the first 30 amino acids of mature protein sequences after removal of predicted signal peptides. **(B–E)** Positional frequencies of YxC, FxC, and WxC motifs along the N-terminal regions of mature secreted proteins in the genera *Blumeria***(B)**, *Erysiphe***(C)**, *Golovinomyces***(D)**, and *Podosphaera***(E)**. Only the first occurrence of the motif in each sequence was considered. Orange, green, and blue bars represent YxC, FxC, and WxC motifs, respectively, while grey bars indicate proteins lacking any of the three motifs.

To characterize N-terminal Y/F/WxC motifs in powdery mildew secretomes, we scanned the first 30 amino acids of mature protein sequences (after removal of predicted signal peptides) for the first occurrence of YxC, FxC, or WxC motifs. Motif counts and proportions were summarized for each isolate (**Figure 4A**; **Supplementary Table 5**), and the positional distribution of motif start sites was characterized at the genus-level for *Blumeria*, *Erysiphe*, *Golovinomyces*, and *Podosphaera* (**Figures 4B–E**; **Supplementary Table 6**).

Across the 26 isolates, Y/F/WxC motif-containing proteins occurred more frequently in *Blumeria* secretomes relative to other powdery mildew genera (**Figure 4A**; **Supplementary Table 5**). In *Blumeria*, 38.8–51.8% of secretome candidates carried at least one N-terminal Y/F/WxC motif within the first 30 residues of the mature sequence, indicating that motif-containing proteins constitute a major fraction of the *Blumeria* secretome. By contrast, motif-containing proteins were less frequent in dicot-infecting genera, with median proportions of 8.5% in *Erysiphe*, 5.6% in *Golovinomyces*, 7.8% in *Podosphaera*, and 2.3% in *Parauncinula* (**Supplementary Table 5**). Motif type composition also differed among genera. *Blumeria* secretomes contained large numbers of both YxC and FxC motifs and a smaller subset of WxC motifs, whereas WxC motifs were absent from *Erysiphe* and were detected only sporadically in *Golovinomyces* and *Podosphaera* (**Figure 4A**; **Supplementary Table 5**).

Motif positions within the N-terminal region of mature proteins further supported genus-associated differences (**Figures 4B–E**; **Supplementary Table 6**). In *Blumeria*, YxC motifs showed a pronounced positional bias, peaking at position 4 of the mature sequence (313 occurrences) (**Figure 4B**). FxC motifs displayed a distinct positional pattern, with prominent peaks at position 17 (192 occurrences) and position 4 (121 occurrences), while WxC motifs were comparatively rare but also showed their highest frequency at position 4 (34 occurrences) (**Figure 4B**). In *Erysiphe*, YxC motifs were most frequently observed at position 16 (23 occurrences) and position 19 (15 occurrences), and only YxC and FxC motifs were detected (**Figure 4C**). *Golovinomyces* exhibited low overall motif counts, with YxC motifs most frequently observed at position 27 (8 occurrences) and WxC motifs primarily at position 7 (3 occurrences) (**Figure 4D**). *Podosphaera* also showed low motif counts, with YxC motifs most frequent at position 16 (19 occurrences) and WxC motifs detected at only a few positions (**Figure 4E**).

Together, these results indicate that N-terminal Y/F/WxC motifs are more frequent in *Blumeria* secretomes and display a genus-specific positional bias in mature protein sequences. This genus-associated motif distribution represents a sequence-based pattern that is consistent with orthogroup-level comparisons and supports subsequent analyses of conserved versus lineage-associated secretome diversification.

### 3.5 Predicted subcellular targeting and functional annotation landscape of powdery mildew secretome candidates

**Figure 5:**
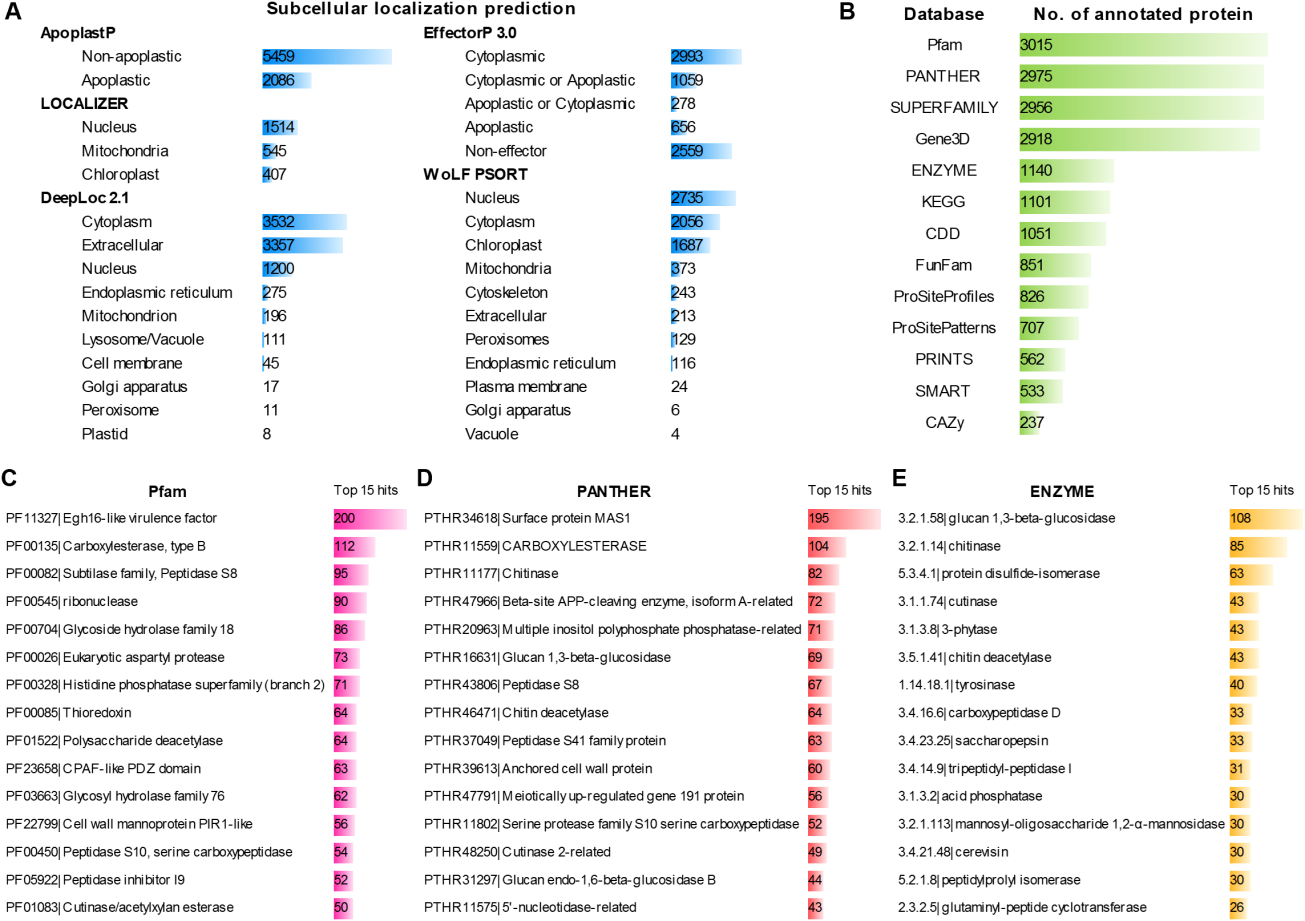
Statistical analysis of subcellular targeting and functional characteristics of powdery mildew secretome candidates. **(A)** Distribution of secretome candidates among different predicted subcellular localizations based on multiple localization prediction tools. **(B)** Number of secretome candidates with functional annotations derived from sequence similarity searches against different protein and functional databases. **(C–E)** Summary of the top 15 functional annotation categories of secretome candidates based on protein domains (Pfam), protein families (PANTHER), and enzymatic activities (ENZYME), respectively.

To summarize predicted targeting properties and functional characteristics of the 7,545 powdery mildew secretome candidates, we integrated subcellular localization predictions from multiple tools and sequence-based functional annotations derived from diverse protein databases (**Figure 5**; **Supplementary Table 3**). Subcellular localization predictions revealed a broad distribution of potential host- and extracellular-associated targeting outcomes (**Figure 5A**). ApoplastP classified 2,086 secretome candidates as apoplastic and 5,459 as non-apoplastic. EffectorP 3.0 assigned 656 candidates as apoplastic effectors and 2,993 as cytoplasmic effectors, with an additional 1,337 candidates predicted to have ambiguous apoplastic or cytoplasmic localization, while 2,559 candidates were classified as non-effectors. DeepLoc 2.1 predicted large fractions of candidates as cytoplasmic (3,532) or extracellular (3,357), with smaller subsets assigned to the nucleus (1,200), endoplasmic reticulum (275), mitochondrion (196), and other compartments. LOCALIZER predicted nuclear targeting for 1,514 candidates and organellar targeting for a smaller number of proteins, whereas WoLF PSORT assigned a wide range of intracellular localizations, including nucleus (2,735), cytoplasm (2,056), chloroplast (1,687), and mitochondria (373). Because individual tools allow multiple or alternative localization assignments, the total counts within each tool do not sum to the full secretome size.

Functional annotation analyses indicated that 4,148 of the 7,545 powdery mildew secretome candidates could be assigned at least one annotation based on sequence similarity to entries in public protein and functional databases (**Figure 5B**; **Supplementary Table 3**). Accordingly, 3,397 candidates had no detectable matches in the queried annotation resources under the applied criteria. Among the databases queried, Pfam provided annotations for 3,015 candidates, followed by PANTHER (2,975), SUPERFAMILY (2,956), and Gene3D (2,918). Additional annotations were obtained from ENZYME (1,140), KEGG (1,101), and CDD (1,051), while smaller subsets of candidates matched FunFam, ProSiteProfiles, ProSitePatterns, PRINTS, SMART, and CAZy databases, indicating variable annotation coverage across different databases within the powdery mildew secretome. Analysis of the most frequently represented annotation categories provided an overview of commonly annotated functional classes among secretome candidates (**Figures 5C–E**). Pfam annotations were enriched for domains associated with secreted virulence and enzymatic activities, including Egh16-like virulence factors, carboxylesterases, subtilisin-like serine proteases, ribonucleases, and glycoside hydrolases. PANTHER classifications similarly emphasized surface-associated proteins, carboxylesterases, chitinases, glucanases, and multiple protease families. Enzyme classification revealed frequent representation of activities linked to cell wall modification, protein processing, and redox-related processes, such as glucan 1,3-β-glucosidases, chitinases, protein disulfide isomerases, cutinases, and phosphatases. Together, these analyses provide an integrated overview of predicted subcellular targeting and functional annotation characteristics of powdery mildew secretome candidates.

### 3.6 Homology mapping of known powdery mildew effectors identifies broadly conserved effector groups across secretome candidates

**Figure 6:**
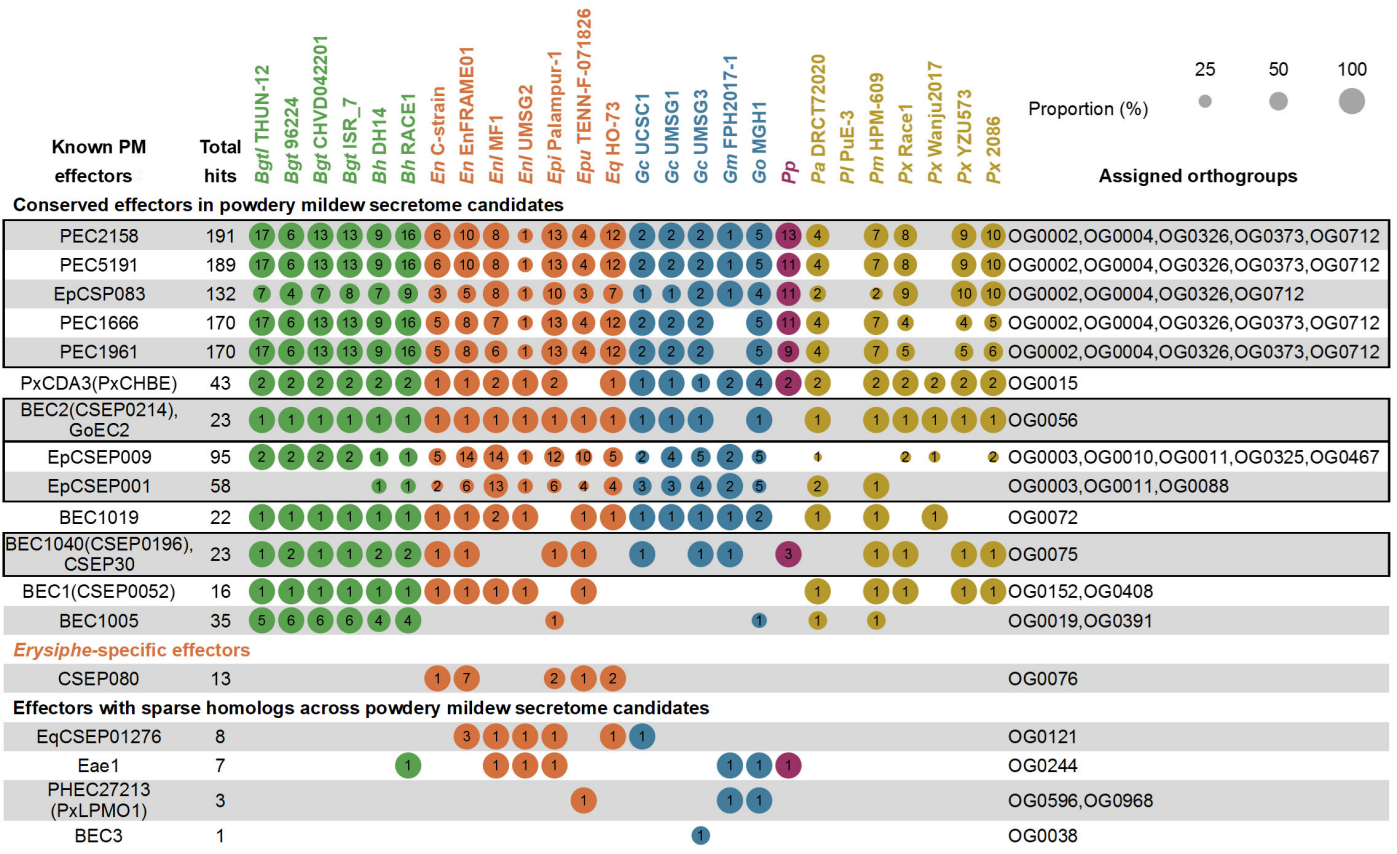
Conservation patterns and lineage specificity of known powdery mildew effectors across secretome candidates from 26 isolates. Bubble plots summarize the distribution of homologs of previously characterized powdery mildew effectors among predicted secretome candidates from 26 powdery mildew isolates. Each row represents a known powdery mildew effector, and columns correspond to individual isolates (abbreviated names are defined in **Supplementary Table 1**). Homologs were identified by BLASTP (E-value < 1e^−5^, query coverage ≥ 50%, identity ≥ 30%). Bubble size reflects the proportion (%) of homologous secretome candidates relative to the total number of candidates assigned to the corresponding orthogroup(s) in each isolate, and numbers within bubbles denote the absolute counts of homologs. Effectors are grouped as conserved (homologs detected in ≥ 10 isolates), *Erysiphe*-specific, or sparsely represented. Assigned orthogroups are shown on the right. Effectors sharing identical homolog distributions are merged into single rows and separated by comma, and black boxes highlight effectors whose homologs are derived from partially overlapping orthogroups.

To link predicted powdery mildew secretome candidates with previously characterized effector repertoires, we compiled 75 known powdery mildew effectors (**Supplementary Table 2**) and searched for homologs among the 7,545 predicted secretome candidates using BLASTP based on the criteria of E-value < 1e^−5^, query coverage ≥ 50%, and sequence identity ≥ 30% (**Supplementary Table 7**). BLASTP thresholds were set to balance sensitivity and specificity: permissive enough to capture divergent homologs across genera, but constrained by E-value, minimum coverage, and minimum identity filters to limit spurious matches. This yielded 2,392 effector-to-candidate homology records corresponding to 1,176 unique secretome candidates with at least one match. Homolog distributions were summarized across 26 isolates and linked to the orthogroups contributing the homolog pools (**Figure 6**).

A dominant signal in **Figure 6** is a broadly conserved, high-abundance effector group represented by PEC2158, PEC5191, PEC1666, PEC1961 from *Podosphaera xanthii* (Martínez-Cruz et al., 2021), and EpCSP083 from *Erysiphe pisi* (Sharma et al., 2019). PEC2158, PEC5191, PEC1666, and PEC1961 were each associated with large homolog pools (170–191 homologs) distributed across 23–24 isolates, with homologs derived from a shared orthogroup combination that includes OG0002, OG0004, OG0326, OG0373, and OG0712. EpCSP083 showed a similarly broad distribution (132 homologs across 23 isolates), with assigned orthogroups overlapping this module and comprising OG0002, OG0004, OG0326, and OG0712. Together, these effectors converge on an overlapping orthogroup-defined candidate set that constitutes one of the most prominent conserved modules in the dataset.

Other conserved effectors mapped to smaller orthogroup-defined homolog pools with consistent multi-isolate representation. PxCDA3(PxCHBE) (Martínez-Cruz et al., 2022) matched 43 homologs across 24 isolates and mapped to OG0015. BEC2(CSEP0214) and GoEC2 (Schmidt et al., 2014; Sabelleck et al., 2025) recovered exactly the same homolog set and mapped to OG0056, with 23 homologs detected across 23 isolates. BEC1040(CSEP0196) (Pliego et al., 2013) and CSEP30 (Li et al., 2024a) also recovered exactly the same homolog set and mapped to OG0075, with 23 homologs detected across 18 isolates (**Figure 6**). In addition, *Blumeria* effectors including BEC1019 (Pliego et al., 2013; Zhang et al., 2019), BEC1(CSEP0052) (Schmidt et al., 2014), and BEC1005 (Pliego et al., 2013) showed broad cross-isolate distributions (**Figure 6**). EpCSEP009 and EpCSEP001 (Sharma et al., 2019) also showed broad cross-isolate representation, with homologs distributed across multiple orthogroups (**Figure 6**).

Outside the conserved set, **Figure 6** includes restricted and sparse distributions. The only *Erysiphe*-specific pattern was CSEP080, which was detected exclusively in *Erysiphe* isolates with 13 homologs. EqCSEP01276 was largely conserved within *Erysiphe* and additionally showed a single homolog in *Golovinomyces cichoracearum* UCSC1. Several other effectors showed low homolog counts and limited isolate coverage (**Figure 6**).

Taken together, **Figure 6** reveals a conserved effector component dominated by a small number of high-abundance orthogroup modules, as well as restricted and sparse patterns. These distinct distribution classes provide a clear contrast to the *Blumeria*-only effector families presented next, where homologs are confined to the monocot-associated lineage.

### 3.7 *Blumeria*-specific effector families reveal lineage-restricted expansion modules in powdery mildew secretomes

**Figure 7:**
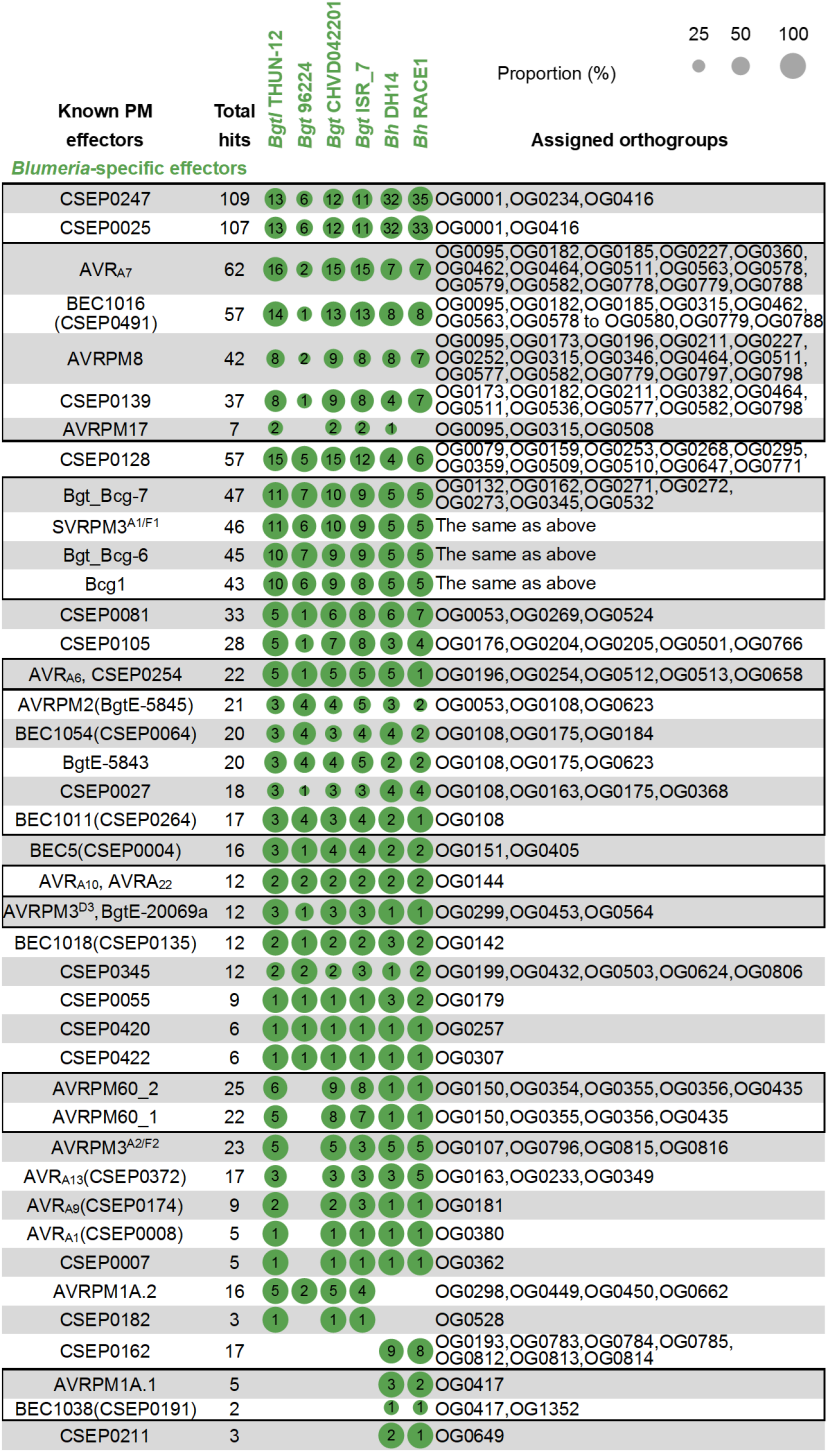
*Blumeria-*specific effector families within predicted secretome candidates. Bubble plots summarize homologs of previously characterized powdery mildew effectors that were detected exclusively in *Blumeria* secretomes following BLASTP searches using the same criteria as in **Figure 6**. Bubble size reflects the proportion (%) of homologous secretome candidates relative to the total number of candidates assigned to the corresponding orthogroup(s) in each isolate, and numbers within bubbles denote the absolute counts of homologs. Assigned orthogroups are shown on the right. Effectors sharing identical homolog distributions are merged into single rows and separated by comma, and black boxes highlight effectors whose homologs are derived from partially overlapping orthogroups.

To resolve lineage-restricted effector families within *Blumeria*, we focused on known powdery mildew effectors whose homologs were detected exclusively in *Blumeria* secretomes using BLASTP based on the criteria of E-value < 1e^−5^, query coverage ≥ 50%, and sequence identity ≥ 30%. **Figure 7** summarizes these *Blumeria*-only homology patterns across the six *Blumeria* isolates and links each effector to the orthogroups contributing its homolog pool.

The largest *Blumeria*-restricted module comprised CSEP0247 (109 homologs) and CSEP0025 (107 homologs), both detected across all six *Blumeria* isolates (**Figure 7**). These two effectors mapped to a strongly overlapping orthogroup set centered on OG0001 and OG0416, with CSEP0247 also showing homologs assigned to OG0234, indicating a shared OG0001-associated candidate pool with a small orthogroup extension in CSEP0247. Within this module, homolog counts were consistently highest in the two *B. hordei* isolates (32–35 for CSEP0247; 32–33 for CSEP0025) relative to the four *B. graminis* isolates (6–13). BUSCO completeness scores for all six *Blumeria* proteomes were high (*B. hordei*: 96.3–98.8%; *B. graminis*: 88.5–93.9%; **Supplementary Table 1**), suggesting that the observed count differences are unlikely to stem from major annotation gaps. Moreover, orthogroup-based clustering inherently groups sequences by homology rather than absolute gene counts, providing a robust framework for comparative analysis. Together, these observations suggest lineage-specific quantitative differences within the genus, with apparent expansion of these effector families in *B. hordei* relative to *B. graminis*.

A second dominant pattern was represented by AVR_A7_ (62 homologs) and BEC1016 (CSEP0491) (57 homologs), which were associated with a shared multi-orthogroup pool and closely related isolate-wise distributions (**Figure 7**). Related patterns were also observed for AVRPM8 (42 homologs) and CSEP0139 (37 homologs), supporting additional mid-sized *Blumeria*-restricted families connected to overlapping candidate pools.

Additional expanded families were represented by effectors with substantial homolog pools and defined orthogroup associations, including CSEP0128 (57 homologs), CSEP0081 (33 homologs), and CSEP0105 (28 homologs) (**Figure 7**). A further high-coverage module comprised Bgt_Bcg-7, SVRPM3^A1/F1^, Bgt_Bcg-6, and Bcg1 (43–47 homologs each), all mapping to the same orthogroup combination and showing highly concordant isolate-wise distributions (**Figure 7**). Several effectors showed identical homolog sets and identical orthogroup assignments, indicating convergence on the same homolog pool. This included AVR_A6_ with CSEP0254 (22 homologs), AVR_A10_ with AVR_A22_ (12 homologs), and AVRPM3^D3^ with BgtE-20069a (12 homologs) (**Figure 7**).

Together, **Figure 7** shows that *Blumeria*-specific effector families are dominated by a small number of high-abundance, orthogroup-linked modules, accompanied by multiple mid-sized families with overlapping or identical homolog pools. This organization contrasts with the cross-genus conserved modules in **Figure 6** and highlights lineage-restricted homology patterns concentrated within *Blumeria*.

### 3.8 Proteome-wide effector homology reveals two broadly conserved core effectors EqCmu and EqPdt

Because six reported powdery mildew effectors (AVR_k1_, Bh9o9, ROPIP1, EqCmu, EqIsc1, and EqPdt) lack predicted signal peptides, searches restricted to the 7,545 secretome candidates cannot capture their full distribution. We therefore performed BLASTP searches against the complete proteome dataset from 26 isolates (219,897 proteins) using the same criteria (E-value < 1e^−5^, query coverage ≥ 50%, identity ≥ 30%) and obtained 389 proteome-wide homologous hits across the six effectors (**Supplementary Table 8**). For comparison, these six effectors were also searched against the predicted secretome candidates, and secretome- versus proteome-level hit summaries are integrated in **Table 1**.

**Table 1:** Summary of BLASTP homologous hits for six non-canonical powdery mildew effectors across predicted secretomes and whole proteomes of 26 isolates.

Effector name	Isolates coverage	Total hits	*Bgtl* THUN-12	*Bgt* 96224	*Bgt* CHVD042201	*Bgt* ISR_7	*Bh* DH14	*Bh* RACE1	*En* C-strain	*En* EnFRAME01	*Enl* MF1	*Enl* UMSG2	*Epi* Palampur-1	*Epu* TENN-F-071826	*Eq* HO-73	*Gc* UCSC1	*Gc* UMSG1	*Gc* UMSG3	*Gm* FPH2017-1	*Go* MGH1	*Pp*	*Pa* DRCT72020	*Pl* PuE-3	*Pm* HPM-609	*Px* Race1	*Px* Wanju2017	*Px* YZU573	*Px* 2086
EqCmu	25	27	1	1	1	1	1	1	1	1	1	1	1	1	1	1	1	1	2	2	1	1	0	1	1	1	1	1
EqPdt	25	27	1	1	1	1	1	1	1	1	1	1	1	0	1	1	1	1	1	3	1	1	1	1	1	1	1	1
EqIsc1	9	9	0	0	0	0	0	0	1	1	1	1	1	1	1	0	0	0	1	0	1	0	0	0	0	0	0	0
AVR_k1_	8	222	0	0	0	0	159	0	18	1	0	1	0	1	0	1	0	0	0	0	0	28	0	0	0	0	0	13
Bh9o9	6	100 (26)	21 (7)	13 (3)	21 (7)	19 (7)	11 (1)	15 (1)	0	0	0	0	0	0	0	0	0	0	0	0	0	0	0	0	0	0	0	0
ROPIP1	3	4	0	0	1	2	0	1	0	0	0	0	0	0	0	0	0	0	0	0	0	0	0	0	0	0	0	0

*Numbers in parentheses indicate the count of Bh9o9 homologs in each isolate secretome.

Proteome-wide mapping revealed lineage-restricted versus broadly conserved non-canonical patterns. Bh9o9 yielded homologous hits in the predicted secretome candidates and showed a larger set of homologs when searched against whole proteomes; in both searches, all Bh9o9 homologs were confined to *Blumeria* in this dataset (**Table 1**). ROPIP1 (4 hits) was detected only in *Blumeria* proteomes, whereas EqIsc1 yielded nine hits that were largely concentrated in *Erysiphe* (**Table 1**). In contrast, EqCmu and EqPdt showed the broadest conservation, with 27 homologous hits detected for each effector and presence in 25 of the 26 proteomes spanning multiple genera (**Table 1**). EqCmu was absent only from *Podosphaera leucotricha* PuE-3, and EqPdt was absent only from *Erysiphe pulchra* TENN-F-071826. The observation that whole-proteome searches recovered more homologs than secretome-only searches underscore a key limitation of signal peptide-based effector discovery: non-canonical effectors lacking signal peptides are systematically excluded from conventional secretome predictions. This is evident for Bh9o9, whose additional homologs may represent variants secreted via alternative pathways or retained as non-secreted paralogs. More importantly, EqCmu and EqPdt are broadly conserved across 25 of 26 proteomes, despite their absence from many predicted secretomes. This widespread conservation reinforces their status as core effectors and highlights the need to complement secretome-focused analyses with proteome-wide searches.

EqCmu and EqPdt homologs not only retained high sequence conservation in multiple sequence alignments (**Supplementary Figures 2**, **3**), but also exhibited high structural similarity to their references (**Supplementary Table 9**; **Supplementary Figures 4**, **5**). Together, their near-universal retention across powdery mildew proteomes and their demonstrated cooperative suppression of host salicylic acid signaling (He et al., 2021; Liu et al., 2025) support EqCmu and EqPdt as two conserved core effectors in powdery mildew fungi.

## 4 Discussion

Powdery mildew fungi are obligate biotrophs that depend on secreted proteins to colonize living host tissues and sustain infection. This study provides a unified sequence-based comparison of predicted powdery mildew secretomes across five genera and 26 isolates, integrating orthogroup analysis, N-terminal Y/F/WxC motif screening, predicted subcellular localization, functional annotation, and homology mapping to known powdery mildew effectors (**Figure 1A**). In this dataset, monocot-associated infection is represented by *Blumeria* lineages, whereas dicot-associated infection spans *Erysiphe*, *Golovinomyces*, *Parauncinula*, and *Podosphaera*. Three principal findings are evident. First, powdery mildew secretomes contain a conserved shared component across genera, while substantial diversification occurs at both genus and isolate levels. Second, the frequent occurrence and positional preference for N-terminal Y/F/WxC motifs characterize *Blumeria* secretomes, reinforcing a distinct sequence signature associated with this *Blumeria*-linked (monocot-associated) lineage relative to dicot-associated genera. Third, homology searches across both secretome candidates and whole proteomes highlight multiple conserved, representative effector groups spanning diverse powdery mildew lineages, with EqCmu and EqPdt serving as two prominent examples supported by their broad occurrence across powdery mildew proteomes, high sequence and structural conservation, and established roles in suppressing host salicylic acid signaling (He et al., 2021; Liu et al., 2025).

An initial insight from our comparative secretome and orthogroup analyses is that powdery mildew secretomes comprise a recurrently detected shared core coupled with pronounced lineage-specific expansions. Secretome size ranged widely among isolates (**Figure 1B**). In particular, *Blumeria* isolates consistently showed larger secretomes than dicot-infecting genera, and this difference was also evident in orthogroup distributions and in the relative proportions of shared versus lineage-restricted orthogroups (**Figure 2A**). Shared orthogroups are relatively consistent across isolates, indicating that a set of conserved secreted protein families is maintained across powdery mildew lineages despite marked variation in total secretome size. Beyond the broad shared category, three orthogroups (OG0007, OG0031, and OG0047) were present in all 26 isolates (**Supplementary Table 4**). Annotation patterns suggest potentially conserved functions, including glycoside hydrolase family 47/mannosyl-oligosaccharide 1,2-alpha-mannosidase-related features for OG0031 and lytic polysaccharide monooxygenase auxiliary activity family 9-related features for OG0047 (**Supplementary Table 3**). Under the applied homology criteria, these orthogroups did not overlap with the currently compiled known-effector homolog set, indicating that broadly maintained secretome modules may include conserved but still functionally undercharacterized components.

*Blumeria* showed a strong accumulation of genus-specific orthogroups, whereas several dicot-associated isolates possessed higher numbers of isolate-specific orthogroups, indicating that diversification acts at different evolutionary scales among powdery mildew lineages (**Figure 2A**). PCA primarily resolved genus-level clusters; because all monocot-associated isolates are *Blumeria*, the apparent host-associated separation is partially confounded by genus (**Figure 2B**). Functional annotations provide biological insight into these orthogroup-level patterns. **Figure 2C** illustrates that genus-level divergence is not only quantitative (**Figure 2A**) but also compositional. The selected orthogroups represent an annotation-informed subset chosen from abundant genus-specific groups, together with annotated dicot-infecting shared groups excluding *Blumeria*, and are intended to exemplify lineage-associated modules rather than provide an exhaustive inventory. Within this representative panel, multiple genera are associated with ribonuclease-related orthogroups, whereas *Podosphaera* is represented by an Egh16-like virulence-associated module; additionally, dicot-infecting shared groups include small secreted proteins, thioredoxin reductase 1-related proteins, and protease/hydrolase-associated families. These compositional differences are consistent with genus-level separation in PCA (**Figure 2B**) and with broader functional annotation trends (**Figures 5C–E**). In addition, the frequent annotation of proteases, hydrolases, and carbohydrate-active enzymes (**Figures 5C–E**) is consistent with secretome repertoires that include not only host-interacting effectors but also enzymes likely involved in modifying the host interface, acquiring nutrients, or metabolizing host-derived substrates. At present, these annotation-linked interpretations are hypothesis-level inferences from sequence-based evidence rather than direct functional assignments. Nonetheless, they provide a biologically informative framework for prioritizing candidates for validation. However, only 4,148 of the 7,545 secretome candidates (55.0%) received at least one database-supported annotation, leaving a substantial fraction functionally uncharacterized despite broad database querying (**Supplementary Table 3**). This large unresolved component may reflect the rapid sequence diversification of secreted proteins in powdery mildews. Together, these results showed that powdery mildew secretomes retain a conserved shared component and prevalent functional classes, while lineage-specific expansions introduce distinct orthogroups that contribute to the divergent patterns observed between *Blumeria* (the monocot-associated genus in this dataset) and dicot-associated lineages.

We further examined sequence attributes of the secretome candidates and found a prevalence of compact proteins, a positive association between cysteine richness and predicted apoplastic localization, and a lineage-specific pattern of higher N-terminal Y/F/WxC motif frequency in *Blumeria*. Most mature proteins were short, with ~50% under 200 amino acids and >75% under 400 amino acids (**Figure 3A**), a size distribution typical of effector-like fungal secreted proteins. Cysteine content ranged mainly from low to moderate, with only a small proportion of candidates classified as cysteine-rich (**Figure 3B**). Notably, the predicted likelihood of apoplastic localization increased with cysteine content in broader genera (**Figure 3B**; **Supplementary Figure 1**), supporting the role of disulfide bonds in stabilizing proteins in the extracellular space. N-terminal motif distributions further separated *Blumeria* from dicot-infecting genera. Y/F/WxC motifs were abundant in *Blumeria* secretomes but rare in other lineages (**Figure 4A**). Within *Blumeria*, motif start positions were tightly constrained: YxC and WxC motifs occurred predominantly at mature position 4, whereas FxC motifs peaked at position 17 (**Figure 4B**). No comparable positional clustering was detected in other genera (**Figures 4C–E**), suggesting that this motif organization represents a *Blumeria*-specific signature rather than a general feature of powdery mildew secreted proteins. The higher frequency of these motifs near the mature N-terminus, especially the peak at position 4, aligns with earlier reports of Y/F/WxC motifs in powdery mildew effector candidates linked to haustorial function (Godfrey et al., 2010). However, because motif occurrence in non-*Blumeria* lineages is sparse and lacks comparable positional concentration, motif presence outside *Blumeria* should not be interpreted as evidence of equivalent function without lineage-specific experimental validation.

Homology-based mapping reveals that conserved effector modules in powdery mildews fall into two broad categories: those maintained across multiple genera and those restricted to specific lineages. A prominent cross-genera module comprising PEC2158, PEC5191, PEC1666, PEC1961, and EpCSP083 shares overlapping orthogroup associations (OG0002, OG0004, OG0326, OG0373, OG0712) and is present in 23–24 isolates (**Figure 6**). The convergence of multiple independently reported effectors onto this orthogroup pool suggests a core set of secreted proteins essential for pathogenesis, established before the divergence of monocot- and dicot-associated lineages. Beyond canonical secreted proteins, EqCmu and EqPdt represent the most broadly conserved effectors across powdery mildews, with homologs detected in 25 of 26 proteomes (**Table 1**). Their strong sequence and structural conservation (**Supplementary Figures 2**-**5**), together with their cooperative suppression of host salicylic acid signaling (He et al., 2021; Liu et al., 2025), positions them as core virulence factors with an ancient and conserved function in host immune manipulation. In contrast, lineage-restricted effector families reveal distinct evolutionary trajectories. *Erysiphe* maintains a small set of genus-associated effectors including CSEP080, EqCSEP01276, and EqIsc1, whereas *Blumeria* has undergone extensive lineage-specific expansions of multiple effector families (**Figure 7**). Together, these patterns suggest a model in which powdery mildew secretomes are built upon a shared core of conserved effectors that likely target fundamental plant processes. Lineage-specific diversification, particularly the pronounced expansions observed in *Blumeria*, reflects adaptation to distinct host environments and the evolutionary pressures imposed by host immunity.

Genus-associated homology patterns further indicate lineage-linked diversification. In *Erysiphe*, the clearest genus-restricted effector is CSEP080 (Mu et al., 2023), detected only in *Erysiphe* isolates here (**Figure 6**). EqCSEP01276 (Li et al., 2020) is largely conserved within *Erysiphe* with a single cross-genus hit under the applied criteria (**Figure 6**), and proteome-wide searches show EqIsc1 (Yin et al., 2024) homologs largely concentrated in *Erysiphe* (**Table 1**). Importantly, the comparative signal extends beyond *Blumeria* expansion. Non-*Blumeria* genera retain the shared core component but appear to weight different accessory modules, suggesting that successful host colonization can be achieved through lineage-specific optimization of smaller repertoires rather than expansion alone. In *Blumeria*, multiple effector families are restricted to this genus and associate with distinct orthogroup combinations (**Figure 7**). The most expanded module comprises CSEP0247 and CSEP0025 (Aguilar et al., 2016), centered on OG0001 and OG0416 with an additional OG0234 extension for CSEP0247 and higher homolog counts in *B. hordei* than *B. graminis* (**Figure 7**). Further *Blumeria*-restricted families include the AVR_A7_ (Saur et al., 2019) and BEC1016(CSEP0491) (Li et al., 2024b) module, with additional mid-sized patterns represented by AVRPM8 (Kunz et al., 2023) and CSEP0139 (**Figure 7**). Several wheat and barley powdery mildew avirulence-associated effectors converge on shared homolog pools with concordant isolate-wise distributions, including Bgt_Bcg-7, SVRPM3^A1/F1^, Bgt_Bcg-6, and Bcg1 (Bourras et al., 2015; Parlange et al., 2015; Schaefer et al., 2020) (**Figure 7**). Identical homolog sets were also observed for AVR_A6_ with CSEP0254 (Ahmed et al., 2016; Bauer et al., 2021), AVR_A10_ with AVR_A22_ (Ridout et al., 2006; Nowara et al., 2010; Amselem et al., 2015; Saur et al., 2019), and AVRPM3^D3^ with BgtE-20069a (Bourras et al., 2019; Yan et al., 2025) (**Figure 7**). Consistent with this genus-restricted pattern, Bh9o9 (Weiß et al., 2025) yielded homologous hits in the predicted secretome candidates and showed a broader set of homologs when searched against whole proteomes, whereas ROPIP1 (Nottensteiner et al., 2018) was detected only in *Blumeria* proteomes in this dataset (**Table 1**). Together, these genus-associated patterns show that alongside broadly conserved effector modules, powdery mildew lineages also retain phylogenetically restricted effector families, with *Erysiphe* preserving a smaller set of genus-associated effectors and *Blumeria* harboring multiple expanded families linked to specific orthogroup combinations.

This study examines 26 powdery mildew isolates from five genera, representing one of the largest isolate sets for comparative secretome analysis to date. Nevertheless, the study captures only a portion of powdery mildew diversity, as other genera (e.g., *Phyllactinia*, *Leveillula*, and *Sawadaea*) could not be included due to limited genomic or proteomic data. Our analysis is also confined to predicted secretome candidates; determining which candidates function during infection will require evidence of in planta expression and delivery. The scarcity of cross-genus RNA-seq datasets collected under comparable infection stages and experimental conditions further limits systematic expression-based validation. Interpretation of host-association patterns is constrained here, as monocot infection is represented solely by *Blumeria* isolates from wheat and barley. Sampling a broader range of monocot-infecting lineages beyond *Blumeria* will help distinguish host-specific effects from genus-level divergence. Finally, prediction-based secretome definitions may miss unconventional secretion, and experimental validation of secretion, localization, and host activity remains essential to translate sequence-based modules into functional effector repertoires. Despite these limitations, the integrated orthogroup analysis, sequence-feature profiles, predicted subcellular localization, functional annotation summaries, and effector-to-candidate homology mapping presented in this study provide a robust and reusable reference for prioritizing conserved core effector modules and lineage-restricted diversification, thereby offering a strategic foundation for future mechanistic investigations into powdery mildew pathogenesis and host adaptation.

## 5 Conclusions

Using a unified sequence−based analytical pipeline across 26 powdery mildew isolates from five genera, this work identifies both a conserved common component of powdery mildew secretomes and extensive, genus− and isolate−specific diversification. In mature secreted proteins, *Blumeria* secretomes show a higher proportion of N-terminal Y/F/WxC motifs and a positional bias, providing a clear sequence signature associated with this lineage. Homology−based mapping of known effectors reveals conserved, high−abundance orthogroup−defined modules present across multiple genera and many isolates, alongside *Blumeria*−specific expanded modules within the monocot−associated lineage examined here. Extending homology searches to whole proteomes further supports EqCmu and EqPdt as two conserved core effectors, consistent with their sequence and structural conservation and previously reported cooperative suppression of host salicylic acid signaling. Together, these findings provide an integrated comparative resource for powdery mildew secretomes and establish a framework for distinguishing conserved core effectors from lineage−specific diversification.

## Data Availability

Publicly available datasets were analyzed in this study. This data can be found here: https://doi.org/10.5281/zenodo.17514294.
