# Supplementary material for: Sequence-based comparative secretome analysis reveals conserved core effectors and host lineage-specific divergence between monocot- and dicot-associated powdery mildew lineages
Source: Front Plant Sci. 2026 Mar 9;17:1783609. doi: 10.3389/fpls.2026.1783609 (PMC13006700; doi:10.3389/fpls.2026.1783609)
Supplement: Supplementary file 2 [file DataSheet2.docx]

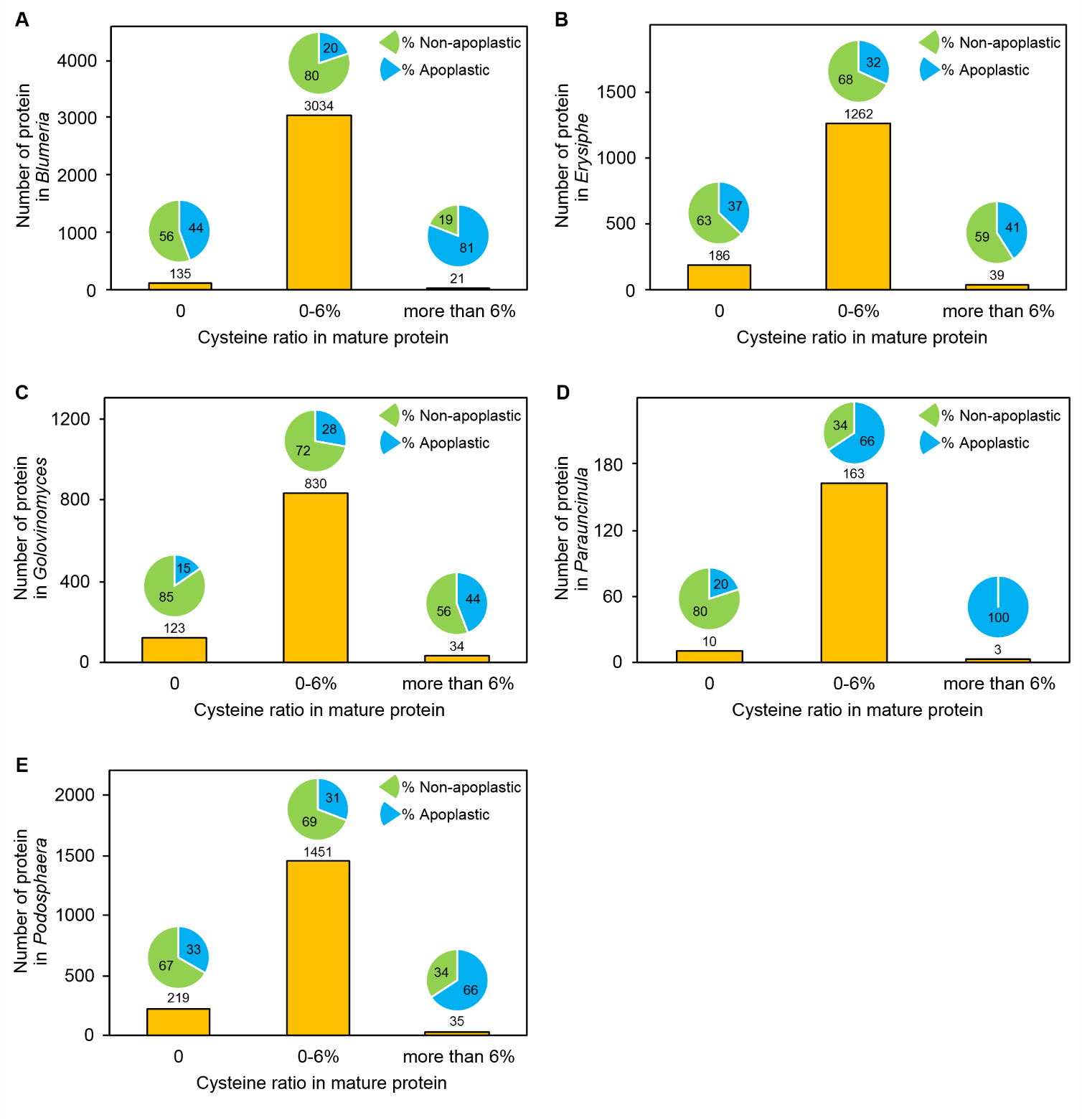


**Supplementary Figure S1.** Genus-stratified distribution of cysteine ratio classes and predicted apoplastic localization in powdery mildew secretome candidates. (**A–E**) Genus-level summaries for *Blumeria* (**A**), *Erysiphe* (**B**), *Golovinomyces* (**C**), *Parauncinula* (**D**), and *Podosphaera* (**E**). Mature-sequence cysteine ratio was calculated as the percentage of cysteine residues after signal peptide removal. Secretome candidates were grouped into three classes: 0% cysteine, >0–6% cysteine, and >6% cysteine. Bars indicate the number of proteins in each cysteine ratio category, while pie charts show the proportions of non-apoplastic and apoplastic proteins predicted by ApoplastP.

**Supplementary Figure S2.** Sequence alignment of the powdery mildew core effector EqCmu and its homologs. Multiple sequence alignment was generated using MUSCLE, and visualized with ESPript v3.2.0. Residue numbering is based on the EqCmu sequence. Conserved regions are boxed in blue. Residue conservation is indicated by red shading (strict identity) and red characters. Gaps introduced for optimal alignment are represented by dashes. The secondary structure elements shown above the alignment correspond to the EqCmu structure predicted by AlphaFold 3, with the following labels: α, α-helices; η, 3_10_-helices; TT, strict β-turns.


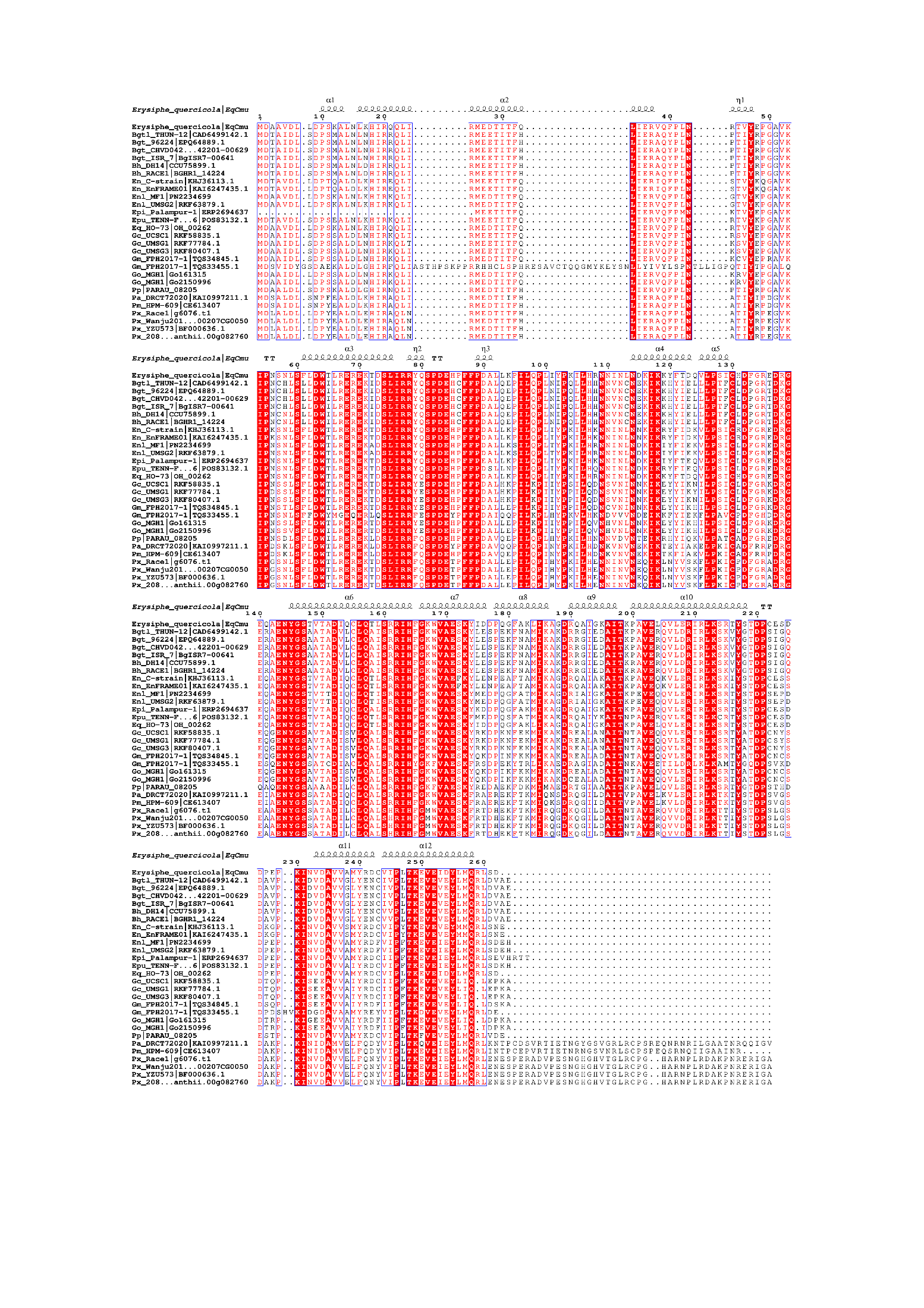


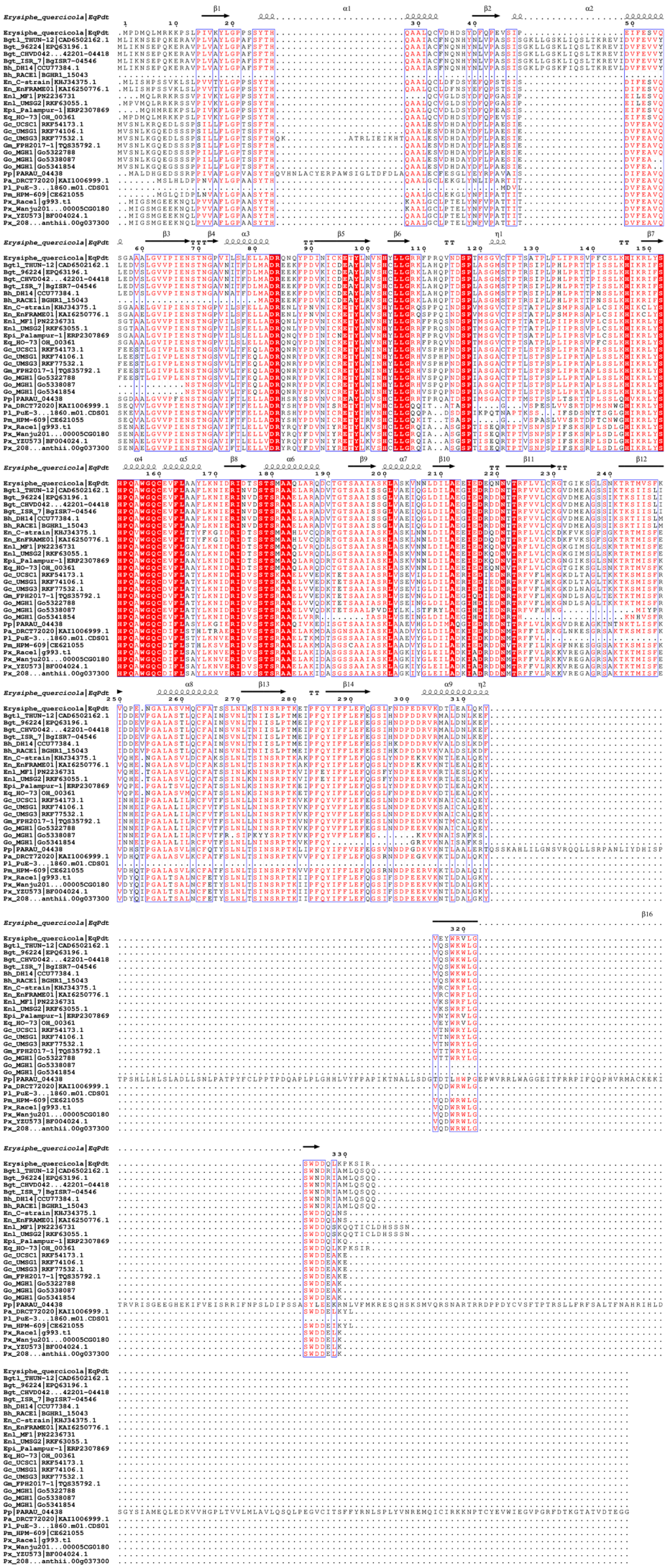


**Supplementary Figure S3.** Sequence alignment of the powdery mildew core effector EqPdt and its homologs. Multiple sequence alignment was generated using MUSCLE, and visualized with ESPript v3.2.0. Residue numbering is based on the EqPdt sequence. Conserved regions are boxed in blue. Residue conservation is indicated by red shading (strict identity) and red characters. Gaps introduced for optimal alignment are represented by dashes. The secondary structure elements shown above the alignment correspond to the EqPdt structure predicted by AlphaFold 3, with the following labels: α, α-helices; η, 3_10_-helices; arrows, β-strands; TT, strict β-turns; TTT, strict α-turns.


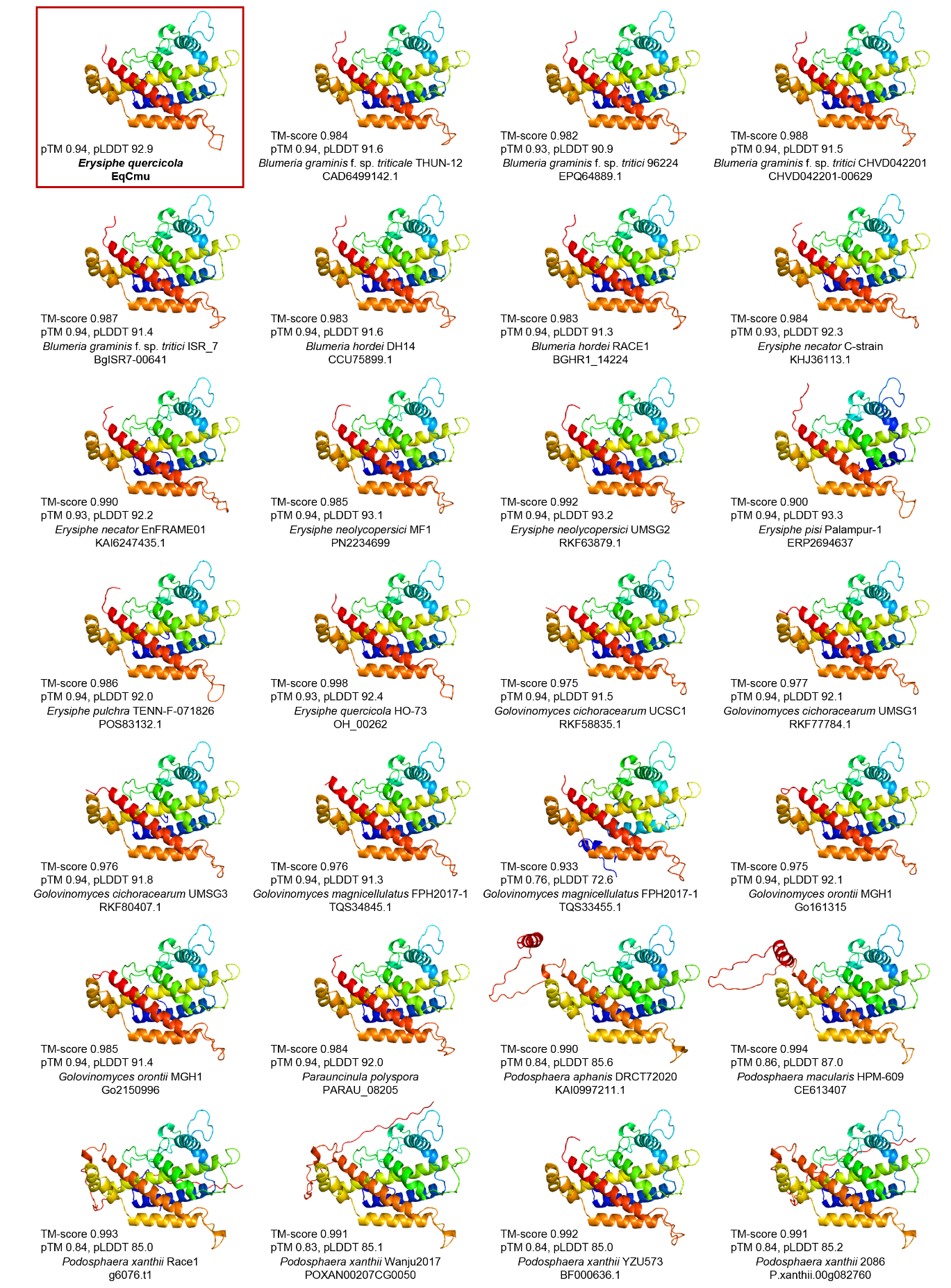


**Supplementary Figure S4. Structural comparison of EqCmu and its homologs. The core effector EqCmu (top-left, red box) and its 27 homologs are shown as structural models predicted by AlphaFold 3. Beneath each model, the predicted pTM (predicted template modeling) and pLDDT (predicted local distance difference test) scores are listed alongside the corresponding powdery mildew isolate and protein ID. The structural similarity between each homolog and EqCmu was quantified using TM-score via US-align, with all scores normalized by the length of EqCmu.**


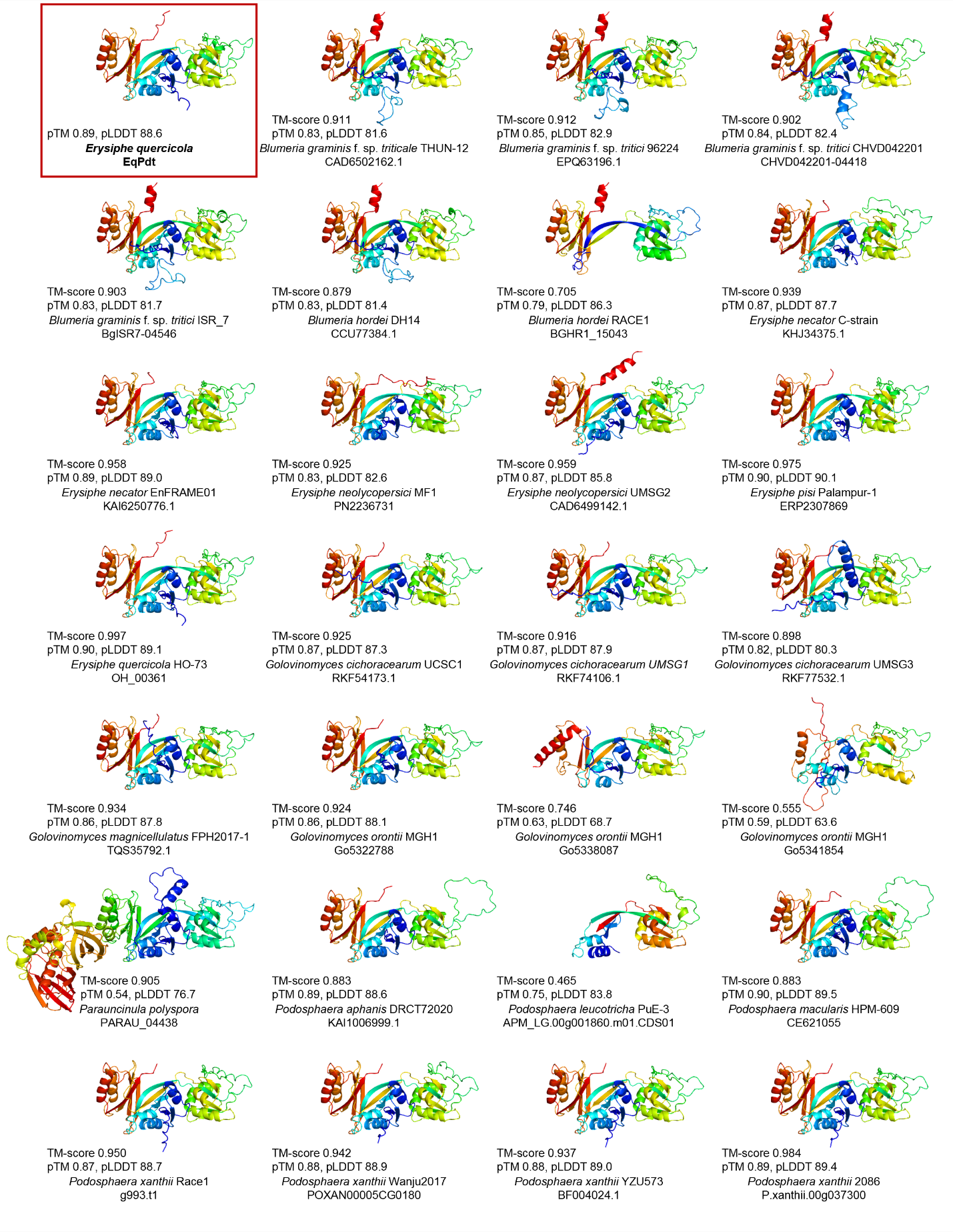


**Supplementary Figure S5. Structural comparison of EqPdt and its homologs. The core effector EqPdt (top-left, red box) and its 27 homologs are shown as structural models predicted by AlphaFold 3. Beneath each model, the predicted pTM (predicted template modeling) and pLDDT (predicted local distance difference test) scores are listed alongside the corresponding powdery mildew isolate and protein ID. The structural similarity between each homolog and EqPdt was quantified using TM-score via US-align, with all scores normalized by the length of EqPdt.**
